# The roles of hybridization and habitat fragmentation in the evolution of Brazil’s enigmatic longwing butterflies, *Heliconius nattereri* and *H. hermathena*

**DOI:** 10.1186/s12915-020-00797-1

**Published:** 2020-07-03

**Authors:** Darli Massardo, Nicholas W. VanKuren, Sumitha Nallu, Renato R. Ramos, Pedro G. Ribeiro, Karina L. Silva-Brandão, Marcelo M. Brandão, Marília B. Lion, André V. L. Freitas, Márcio Z. Cardoso, Marcus R. Kronforst

**Affiliations:** 1 Department of Ecology & Evolution, The University of Chicago, Chicago, IL USA; 2 Departamento de Biologia Animal e Museu de Zoologia, Instituto de Biologia, Universidade Estadual de Campinas, Campinas, SP Brazil; 3 Centro de Biologia Molecular e Engenharia Genética, Universidade Estadual de Campinas, Campinas, SP Brazil; 4 Centro de Ciências Naturais e Humanas, Universidade Federal do ABC, Santo André, SP Brazil; 5 Departamento de Ecologia, Universidade Federal do Rio Grande do Norte, Natal, RN Brazil

**Keywords:** *Heliconius*, Hybridization, Introgression, Mutation load, Mimicry, Phylogenetics

## Abstract

**Background:**

*Heliconius* butterflies are widely distributed across the Neotropics and have evolved a stunning array of wing color patterns that mediate Müllerian mimicry and mating behavior. Their rapid radiation has been strongly influenced by hybridization, which has created new species and allowed sharing of color patterning alleles between mimetic species pairs. While these processes have frequently been observed in widespread species with contiguous distributions, many *Heliconius* species inhabit patchy or rare habitats that may strongly influence the origin and spread of species and color patterns. Here, we assess the effects of historical population fragmentation and unique biology on the origins, genetic health, and color pattern evolution of two rare and sparsely distributed Brazilian butterflies, *Heliconius hermathena* and *Heliconius nattereri*.

**Results:**

We assembled genomes and re-sequenced whole genomes of eight *H. nattereri* and 71 *H. hermathena* individuals. These species harbor little genetic diversity, skewed site frequency spectra, and high deleterious mutation loads consistent with recent population bottlenecks. *Heliconius hermathena* consists of discrete, strongly isolated populations that likely arose from a single population that dispersed after the last glacial maximum. Despite having a unique color pattern combination that suggested a hybrid origin, we found no genome-wide evidence that *H. hermathena* is a hybrid species. However, *H. hermathena* mimicry evolved via introgression, from co-mimetic *Heliconius erato*, of a small genomic region upstream of the color patterning gene *cortex*.

**Conclusions:**

*Heliconius hermathena* and *H. nattereri* population fragmentation, potentially driven by historical climate change and recent deforestation, has significantly reduced the genetic health of these rare species. Our results contribute to a growing body of evidence that introgression of color patterning alleles between co-mimetic species appears to be a general feature of *Heliconius* evolution.

## Background

Butterflies have served as a historically significant model of insect diversity because of the group’s extreme species diversity, containing an estimated 18,000 species, and the even more striking color pattern diversity that defines the subspecies, races, forms, and morphs of many butterfly species. Among butterflies, the Neotropical genus *Heliconius* has attracted attention for centuries because of their bold and highly variable wing color patterns [1–4], as well as their fascinating behavior and natural history, which includes co-evolved relationships with larval host plants, widespread Müllerian mimicry, adult pollen feeding, and pupal-mating behavior [2, 4–6]. Today, researchers are leveraging the natural diversity of *Heliconius* butterflies and genomic tools to characterize the molecular genetic basis of mimicry [7–13] and track genome-wide patterns of differentiation and introgression associated with speciation [14–20]. Yet, for all of the attention, a number of fascinating *Heliconius* species have evaded modern genomic interrogation. Two particularly striking examples are *Heliconius nattereri* and *Heliconius hermathena* from Brazil.

**Fig. 1:**
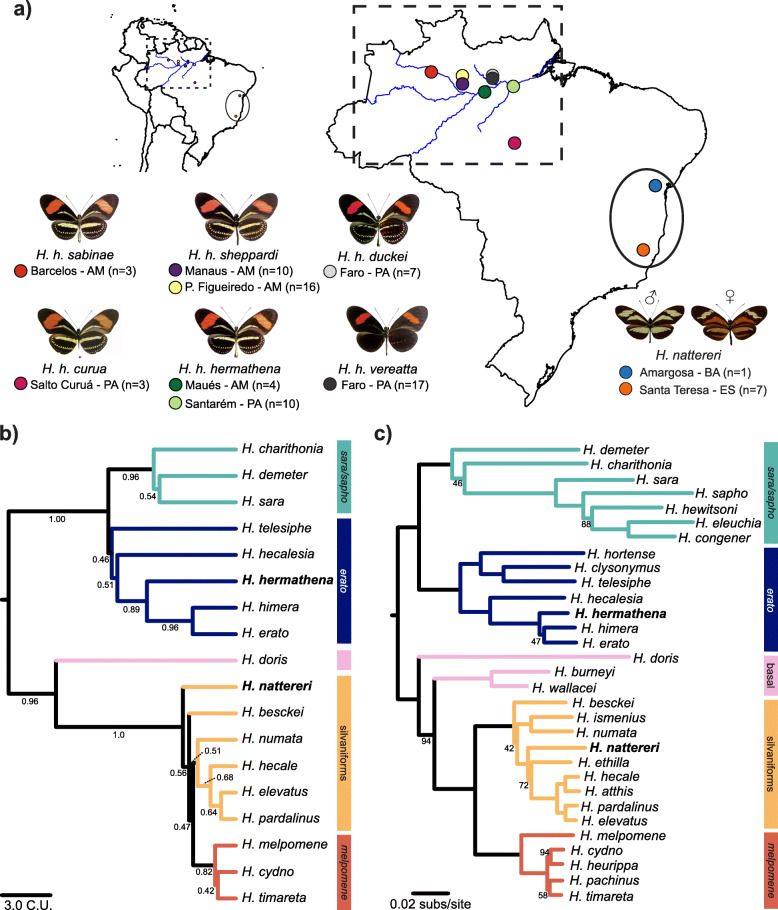
Distribution and phylogenetic relationships of *Heliconius hermathena* and *H. nattereri.***a** Sampling areas and phenotypes of *H. hermathena* subspecies and *H. nattereri*. *H. hermathena* images show dorsal color patterns on the left and ventral on the right. Brazilian states: AM, Amazonas; BA, Bahia; ES, Espírito Santo; PA, Pará. **b** ASTRAL tree constructed from 8674 trees based on 10-kb autosomal windows, rooted with *Eueides tales* (not shown). Quartet scores are shown below branches. C.U., coalescent units. **c** Maximum likelihood tree based on complete mitochondrial DNA sequences. Only branches with less than 100% bootstrap support are labeled. subs/site, substitutions per site. *H. nattereri* images courtesy of AHB Rosa

*Heliconius nattereri* is an exceptionally rare, geographically restricted species found only along a section of Brazil’s Atlantic Forest spanning portions of the states of Espírito Santo and Bahia [21–23]. *Heliconius nattereri* stands out relative to other species in the genus because it is the only *Heliconius* species that is endangered [24] and it is also the only *Heliconius* species with pronounced sexually dimorphic color patterns (Fig. 1). Female *H. nattereri* display a black, yellow, and orange “tiger stripe” pattern that facilitates mimicry with other co-occurring *Heliconius* and Ithomiini species while males display a non-mimetic yellow and black wing pattern. Prior to Keith Brown’s rediscovery of *H. nattereri* in the 1960s [21, 22], this species was only known from approximately 20 pinned specimens in museums around the world. Furthermore, males and females had not been collected together, and because of their pronounced sexual dimorphism in both color pattern and behavior, females had been historically described as a different species, *H. fruhstorferi* Riffarth, 1899. In the 1960s and 1970s, Brown and colleagues extensively surveyed the range of *H. nattereri*, discovering additional localities and specimens, including one large population that they studied intensively for three years. Based on his work, Brown was not optimistic about the long-term future of *H. nattereri* because of its limited and still dwindling habitat and his observations that it seemed to be an “inflexible” and “sensitive” species [22].

Like *H. nattereri*, *H. hermathena* has historically been viewed as an unusual and relictual species [25]. Much of what we know about *H. hermathena* stems from Brown and Benson’s [25] extensive survey, in which they described the systematics, biogeography, natural history, ecology, and behavior of this species. More recently, Seixas et al. [26] described the population ecology of *H. hermathena* based on a 14-month mark-recapture experiment. *Heliconius hermathena* is associated with white sand habitats (*campina* and *campinarana*) throughout the Brazilian Amazon. These patchy, harsh environments produce a network of seven described *H. hermathena* subspecies, including *H. hermathena curua*, which was just described in 2019 [27]. Six *H. hermathena* subspecies display subtle variations on a shared, putatively non-mimetic color pattern (Fig. 1) while the seventh subspecies, *vereatta*, lacks much of the yellow patterning on the fore- and hindwings and is a co-mimic of sympatric *Heliconius erato* and *Heliconius melpomene*. *Heliconius hermathena vereatta* has a limited spatial distribution and is known to freely hybridize with adjacent *Heliconius hermathena duckei* near the town of Faro in the State of Pará [25]. Based on the phenotypic variation among individuals and color pattern frequencies in the field, Brown and Benson [25] inferred that mimicry in *H. hermathena* was controlled by a single locus with a dominant, melanic allele. The genetics of mimicry in *H. hermathena* has not been further explored. The evolutionary origin of *H. hermathena* itself is a topic of interest. The non-mimetic color pattern shared by most *H. hermathena* subspecies appears to be a combination of features from close relatives *H. erato* and *H. charithonia*, and Hewitson [28] reflected this noticeable mixture when naming the species. Today, there are a number of well-characterized instances of introgression and hybrid speciation in *Heliconius* [15, 19, 20, 29], and while *H. hermathena* is considered one possible example of hybrid speciation [30–32], the evolutionary origin of this species remains largely unexplored.

As part of an international collaboration, we have been studying the biology, genomics, and population genetics of Brazil’s endemic, and enigmatic, *Heliconius* fauna. With permission and funding from the Brazilian government, we have performed new surveys of *H. nattereri* [23] and *H. hermathena* [27] populations, collected samples, and performed genomic analyses. Here, we present reference genome sequences and whole-genome resequencing datasets for both species. We use these data to infer their modern population structure and historical demography relative to other well-studied and widespread *Heliconius* species, characterize the genomic consequences of population decline and historical habitat fragmentation, and define the genetic basis of mimicry in *H. hermathena* and trace its evolutionary origin. These data add to a growing list of genomic resources in *Heliconius* [15, 20, 33] but crucially yield novel insight into the biology of two unique but critically understudied species.

## Results

### High-quality reference genome assemblies for *H. hermathena* and *H. nattereri*

We first generated high-quality *H. hermathena* and *H. nattereri* genome assemblies to facilitate phylogenetic and population genomic analyses using Illumina paired-end and mate-pair sequencing (Table 1; Tables S1-S2, Additional file 1; the “Materials and methods” section). The final *H. hermathena* assembly comprised 392 Mb in 1913 scaffolds with an N50 of 560 kb, while the *H. nattereri* assembly comprised 276 Mb in 261 scaffolds with an N50 of 8.8 Mb (Table 1). These values were consistent with the size and heterozygosity estimates of 373 Mb and 0.0057 for *H. hermathena* and 258 Mb and 0.0075 for *H. nattereri*, respectively, from analyses of 21-mer frequencies in the raw sequencing data [34]. Furthermore, both genomes were predicted to be among the most complete and least redundant nymphalid assemblies available based on the presence and completeness of universal single-copy orthologs assayed using BUSCO [35] (Table 1).

**Table 1:** *Heliconius hermathena* and *H. nattereri* genome assembly statistics in comparison with other well-assembled nymphalid genomes

Species^a^	Length (Mb)	No. of scaffolds^b^	N50 (Mb)	BUSCO^c^		
				Comp. (Dup.)	Frag. (%)	Miss. (%)
*Bicyclus anynana*	467	2423	0.66	89.6% (0.7%)	3.9	6.5
*Danaus plexippus*	245	1009	0.73	96.6% (1.9%)	2.3	1.1
*Heliconius charithonia*^d^	316	5162	0.11	94.3% (0.3%)	3.8	1.6
*Heliconius erato demophoon*	383	194	10.69	85.5% (0.7%)	4.5	10.0
*Heliconius erato lativitta*	418	142	5.48	73.4% (0.8%)	5.4	21.2
***Heliconius hermathena***	**392**	**1913**	**0.56**	**96.0% (0.3%)**	**2.4**	**1.6**
*Heliconius melpomene*	275	209	14.31	86.2% (0.4%)	4.1	9.7
***Heliconius nattereri***	**276**	**261**	**8.84**	**97.3% (0.5%)**	**1.2**	**1.5**
*Hypolimnas misippus*	409	1580	1.01	88.9% (0.3%)	3.8	7.3
*Junonia coenia*	586	1101	1.57	96.9% (14.7%)	1.4	1.7
*Limenitis arthemis*	306	737	2.27	81.7% (1.4%)	1.9	16.4
*Melitaea cinxia*	383	5633	0.12	57.1% (0.2%)	11.8	31.1
*Vanessa tameamea*	357	1558	2.99	96.3% (0.4%)	2.2	1.5

^a^Accessions are shown in the “Materials and methods” section

^b^Scaffolds ≥ 5 kb

^c^Calculated using BUSCO v3 and the Endopterygota database (2440 SCOs) from OrthoDB v9

^d^Assembled here, see the “Materials and methods” section

### Phylogenetic placement of *H. hermathena* and *H. nattereri*

*Heliconius* consists of two major clades with unique characteristics: the *erato-sara* clade and the *melpomene*-silvaniform clade (Fig. 1). Morphological studies have placed *H. hermathena* within the *erato-sara* clade [25] and *H. nattereri* within the *melpomene*-silvaniform clade, but molecular phylogenetics results have been unclear about the fine-scale placement of species. Beltrán et al. [30] used four autosomal and four mitochondrial genes while Kozak et al. [36] used 20 autosomal and three mitochondrial genes to infer *Heliconius* species relationships. These studies placed *H. nattereri* and *H. ethilla* as sister species with low statistical support and *H. hermathena* as a polytomy with *H. himera* and *H. erato*. In a recent pre-print, Kozak et al. [37] used genome-wide SNP calls relative to *H. melpomene* to reconstruct the *Heliconius* species tree and found *H. hermathena* as sister to *H. erato* and *H. himera*; they did not include *H. nattereri*. However, hybridization and gene flow are common within the major *Heliconius* clades, confounding estimation of species relationships using standard molecular phylogenetic methods based on few loci or a single reference genome [15, 20, 37].

To resolve the placement of *H. hermathena* and *H. nattereri* within *Heliconius*, we performed whole-genome alignments and reconstructed species trees based on genome-wide data following Edelman et al. [20]. Edelman et al. [20] inferred the relationships among 13 *Heliconius* species by aligning de novo-assembled genomes using the progressiveCactus alignment pipeline [38, 39], inferring gene trees for short non-overlapping aligned regions, and summarizing those gene tree topologies using ASTRAL [40, 41]. This approach therefore largely avoids biases introduced by using a single reference genome or a small number of gene trees and therefore more fully captures the different relationships among different genome regions due to gene flow [20]. Support for each branch in the species tree is calculated as the fraction of gene trees that include particular four-taxon topology (ASTRAL’s quartet score) [40]. We aligned our new reference genomes and a new high-quality *Heliconius charithonia* reference assembly that we generated using publicly available data (Table 1; the “Materials and methods” section) to the multi-species alignment produced by Edelman et al. [20] using progressiveCactus [38, 39]. We then constructed maximum likelihood (ML) trees using autosomal, non-overlapping 10-kb windows and used these trees to infer the *Heliconius* species tree with ASTRAL-iii (Fig. 1; Figure S1–S10, Additional file 2) [38–42]. The final species tree was inferred using 18 *Heliconius* genomes and 8674 windows (Fig. 1b). We corroborated these results with smaller coding and non-coding alignment blocks, where the effects of intra-alignment recombination are limited; within the *melpomene-*silvaniform and *erato-sara* clades separately; and using only Z-linked windows, as the Z is generally more resistant to gene flow and may better represent the true relationships between the species (Figure S2-S10, Additional file 2; the “Materials and methods” section) [20].

All of these analyses placed *H. hermathena* as sister to *H. erato* and *H. himera* (Fig. 1b; Figure S7-S10, Additional file 2). The branches joining these species together and separating *H. hermathena* from the other two had quartet scores > 0.89 in analyses of autosomal and Z-linked windows (Fig. 1b; Figure S8-S9, Additional file 2), and somewhat lower scores when using the shorter coding and non-coding blocks (> 0.54; Figure S7, Additional file 2). These results are consistent with the results of Kozak et al. [37] based on genome-wide SNP data. The primary source of discordance (i.e., low quartet scores) in the *erato-sara* clade is the hybridization event or events between the *H. sara*/*H. demeter* ancestor and *H. hecalesia* described by Kozak et al. [37] and Edelman et al. [20]; this event was reflected by the different relationships between these species in the predominant 10-kb window topologies (Figure S11, Additional file 2) in our analyses.

The placement of *H. nattereri* remained less certain. First, in contrast to Beltrán et al. [30] and Kozak et al. [36], all of our reconstructions based on autosomal loci place *H. nattereri* as an outgroup to the remaining members of the *melpomene-*silvaniform clade (Fig. 1b; Figure S7-S10, Additional file 2). Consistent with previous studies, the *melpomene* clade nested within the silvaniforms, and there was generally low concordance among trees estimated from different regions (Fig. 1b; Figure S7-S8, Additional file 2). Second, species tree reconstructions based on Z-linked windows recovered a clade comprising *H. nattereri*, *H. numata*, and *H. besckei* as an outgroup to the remaining *melpomene*-silvaniform clade species (Figure S9, Additional file 2). Kozak et al. [37] did not include *H. nattereri* but recovered a similar lineage in their analysis of Z-linked markers that included *H. numata*, *H. besckei*, and *H. ismenius*.

Finally, we assembled and analyzed mitochondrial genomes of 33 *Heliconius* species to more directly compare our results with previous studies. We assembled a typical ~ 15-kb contig for 31 of 33 species by extracting and assembling 26 new mitochondrial genomes from publicly available sequencing data using NOVOPlasty and seven reference mitochondrial genomes (Table S3, Additional file 1) [43]. We then inferred species relationships using these 33 sequences and ML (Fig. 1c) [42]. The relationships we found were similar to those recovered by the smaller mtDNA analyses in Beltrán et al. [30] and Kozak et al. [36], with differences only in clades that historically have been difficult to resolve such as in the *melpomene*-silvaniform clade (Fig. 1c). This phylogeny was identical to the one recovered by Kozak et al. [37]. Importantly, *H. hermathena* was inferred to be sister to *H. erato* and *H. himera*. However, in sharp contrast with species tree reconstructions based on genome-wide data, the mtDNA analysis recovered the silvaniforms as a monophyletic clade, with *H. nattereri* nested within.

### Small effective population sizes of *H. nattereri* and *H. hermathena* result in high deleterious mutation loads

Both *H. hermathena* and *H. nattereri* have patchy, limited distributions that make them difficult to find and study in their natural habitats. *Heliconius nattereri* in particular is restricted to a few pockets of Atlantic Forest in a narrow region of eastern Brazil, usually only above ~ 500 m elevation, and is already listed as endangered by the IUCN [21–24]. To better understand the genetic health of these rare species, we estimated their current genetic diversity, historical population sizes, and deleterious mutation loads using population genomic data.

**Fig. 2:**
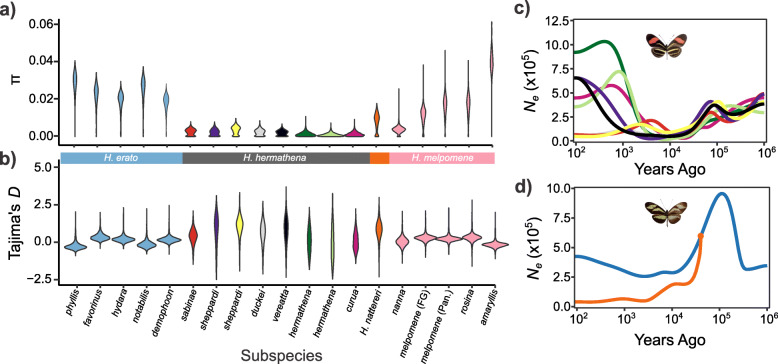
Current and historical effective population sizes in *Heliconius nattereri* and *H. hermathena*. **a** Nucleotide diversity per site (*π*) calculated in non-overlapping 10-kb windows across the autosomes. **b** Tajima’s *D* statistic calculated in 10-kb non-overlapping windows across the autosomes. **c***SMC++* analysis results for *H. hermathena*. **d***SMC++* analysis results for *H. nattereri.* Color schemes follow those in Fig. 1a. Geographical assignments for *H. erato* and *H. melpomene* subspecies are shown in Table S4 (Additional file 1)

We sequenced the complete genomes of eight *H. nattereri* individuals from two locations and 71 *H. hermathena* individuals spanning six subspecies from seven localities (3–19 individuals per site) and used those data to analyze patterns of variation within and between populations (Fig. 1; Table S1, Additional file 1). We first estimated nucleotide diversity per site (*π*) in populations of *H. hermathena*, *H. nattereri*, and their close relatives *H. erato* and *H. melpomene* for comparison (Fig. 2; Tables S4-S5, Additional file 1). *Heliconius erato* and *H. melpomene* are widespread, abundant species that have well-characterized genetic history and population structure (e.g., [14, 17]). While average *π* in *H. melpomene* and *H. erato* was 0.0197 and 0.0251, respectively, consistent with previous estimates [14, 17, 44], *H. nattereri* and *H. hermathena* carry average *π* of only 0.0072 and 0.0047 (Fig. 2; Table S5, Additional file 1). *H. hermathena* populations in particular contain little genetic diversity, with average *π* ranging from only 0.0011–0.0036, consistent with observations of few individuals in any one locality [25]. The species-wide *π* values are consistent with analyses of 21-mers performed during reference genome assemblies above. We estimated that the current effective population size (*N*_*e*_) of *H. nattereri* is ~ 620,000 (327,000–1,385,000), and the current *N*_*e*_ of *H. hermathena* is ~ 405,000 (214,000–904,000; Table S5, Additional file 1) using the measured *H. melpomene* mutation rate of 2.9e−9 per site per generation (*μ*; 95% CI 1.3e−9 to 5.5e−9) [45] and the classic estimator *π* = 4*N*_*e*_*μ* [46]. These estimates should be viewed with caution, however, as the relationship between *π* and *N*_*e*_ assumes that populations are at equilibrium and that variant sites are evolving strictly neutrally, assumptions that are not likely to be true (see below).

Current *N*_*e*_ estimates reflect the harmonic mean of population sizes over recent history. To better understand the recent history of *H. hermathena* and *H. nattereri*, we estimated historical population sizes using the a multi-sample coalescent approach implemented in *SMC++* (Fig. 2) [47]. We found a mixture of *H. hermathena* population size histories (Fig. 2). However, all *H. hermathena* populations were predicted to have been small and followed similar trajectories until ~ 10,000 years ago, when the southern populations *H. h. vereatta* and *H. h. sheppardi* from Manaus expanded quickly. In contrast to *H. hermathena*, we found that *H. nattereri* population sizes reached a peak ~ 100,000 years ago and have declined steadily since. The Santa Teresa population in particular has remained small, *N*_*e*_ ≈ 40,000, for the past 30,000 years. The most recent estimates place the Bahia and Santa Teresa population sizes at 424,000 and 40,000, respectively (Fig. 2; Table S4). The declines in *H. nattereri* and *H. hermathena* coincide with the end of the last glacial maximum, about 12,000 years ago.

Previous studies of *Heliconius* population size histories have only used the single-sample coalescent method implemented in PSMC [48]. However, *SMC++* is more powerful and able to accurately infer more recent population size changes than PSMC [47]. We include PSMC results for comparison in Figure S12 (Additional file 2). The PSMC and *SMC++* results from 10^4^ to 10^6^ years ago are nearly identical.

We consistently found that *H. nattereri* and *H. hermathena N*_*e*_ was only 20–25% that of their more widely distributed relatives *H. melpomene* and *H. erato* (Fig. 2). Small populations are expected to harbor more slightly deleterious alleles due to the strength of genetic drift relative to natural selection, so we expected *H. nattereri* and *H. hermathena* to carry higher deleterious mutation loads than other closely related species with larger effective population sizes. We expected this signal to be especially strong if these species underwent a recent, strong population bottleneck like the one predicted by *SMC++* [49, 50]. We therefore calculated the numbers of species-specific substitutions and the site frequency spectrum of derived mutations as qualitative measures of *H. nattereri* and *H. hermathena* genetic health*.*

**Fig. 3:**
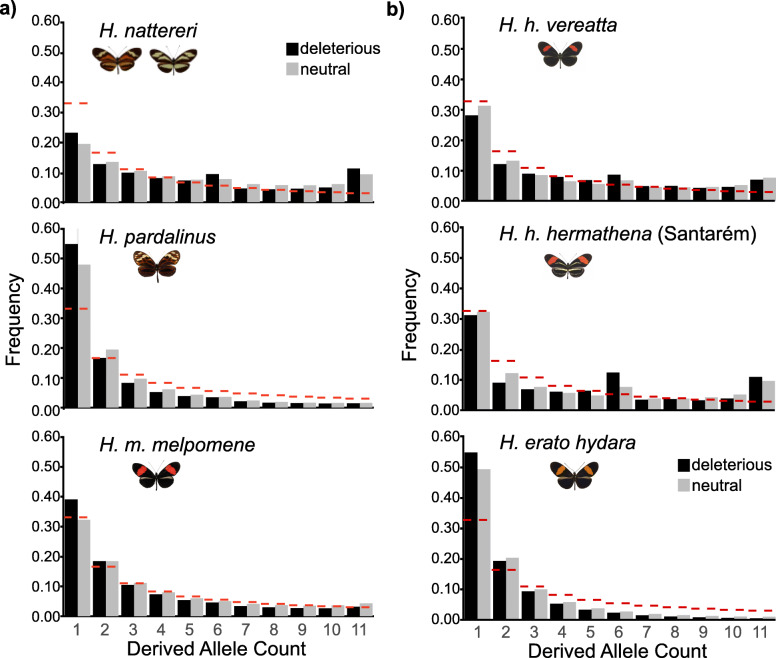
Deleterious mutation loads in *Heliconius nattereri* and *H. hermathena*. **a** Derived allele frequency spectra in the Santa Teresa *H. nattereri* population, its close relative *H. pardalinus*, and *H. melpomene melpomene*. **b** Derived allele frequency spectra from *H. erato* and *H. hermathena* populations with the largest sample sizes. Dashed red lines indicate expected fractions based on the coalescent

We first compared substitutions and the frequency spectrum of derived polymorphisms in *H. nattereri* to those in its close relatives *H. pardalinus* and *H. melpomene* (Fig. 3a). We called SNPs for each species relative to the *H. melpomene* genome, inferred the ancestral state for each site, then inferred the impact of each derived mutation on *H. melpomene* gene models using snpEff [51]. We then calculated the frequency spectra for neutral and deleterious mutations separately (the “Materials and methods” section). *H. nattereri* has accumulated a significantly higher fraction of deleterious substitutions (7.5% of all substitutions) than either *H. pardalinus* (5.9%) or *H. melpomene* (2.0%) since they diverged from their last common ancestor (*χ*_1_^2^ > 1272, *p* < 2e−16 for the three tests; Table S6, Additional file 1). Furthermore, *H. nattereri* harbors an excess of alleles at intermediate and high frequencies, regardless of their impact (Fig. 3). This excess of intermediate and high-frequency alleles further suggests that *H. nattereri* recently underwent a strong population bottleneck, consistent with the *SMC++* analysis (Fig. 2). This bottleneck hypothesis was supported by a high genome-wide average (+ 0.82) and standard deviation (0.66) of Tajima’s *D* statistic in *H. nattereri* (Table S5, Additional file 1) [52]. We did not observe these SFS or Tajima’s *D* patterns in *H. pardalinus* or *H. melpomene*, despite those species carrying similar or higher levels of diversity to *H. nattereri* (0.0077 and 0.015, respectively; Fig. 3). Our results therefore quantify the threatened status of *H. nattereri* at the genetic level.

We found similarly skewed substitution proportions, site frequency spectra, and Tajima’s *D* distributions in *H. hermathena*, suggesting a recent bottlenecks and high deleterious mutation load (Figs. 2 and 3). *H. hermathena* contains an excess of both fixed (*χ*_1_^2^ = 5100, *p* < 2e−16; Table S7, Additional file 1) and intermediate- and high-frequency deleterious alleles relative to *H. erato* (*χ*^2^_10_ > 3,032,371, *p* < 2e−16 for all tests). The strength of the skew was inversely related to current *N*_*e*_ estimates based on *π* (Figs. 2 and 3).

### Strong population structure in *Heliconius hermathena* likely causes small *N*_*e*_

While there is good evidence that *H. nattereri* is a sensitive and rare species, it was less clear why *H. hermathena* exhibits such small population sizes and high deleterious mutation loads. However, *H. hermathena* comprises seven recognized subspecies from white sand habitats (*campina* and *campinarana*) scattered around the Amazon River Basin that few other *Heliconius* species can tolerate [25–27]. This patchy distribution, high habitat fidelity, and observed low dispersal led Brown and Benson [25] to hypothesize that *H. hermathena* was once widespread but recently fragmented by the expansion of the Amazon rainforest after the last glacial maximum, about 12,000 years ago. We therefore tested whether *H. hermathena* population fragmentation was contributing to the population genomic patterns we observed.

**Fig. 4:**
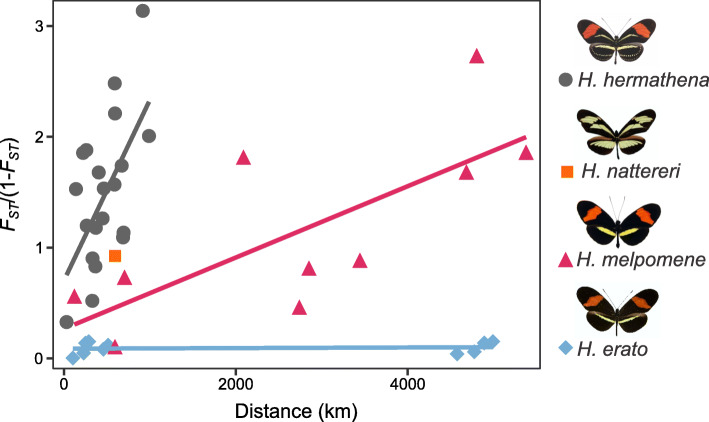
The relationship between genetic distance (*F*_ST_/(1 − *F*_ST_)) and geographical distance for *Heliconius nattereri*, *H. hermathena*, *H. melpomene*, and *H. erato* populations, see Tables S1 and S4 for population and geographical location information (Additional file 1)

We first assayed genetic differentiation between *H. hermathena* populations relative to their widespread relatives using *F*_ST_. Consistent with the hypothesis that their populations are strongly isolated, we found a strong positive correlation between *F*_ST_ and geographical distance between *H. hermathena* populations (Fig. 4; Table S8, Additional file 1). The rate at which *F*_ST_ increases with geographical distance is nearly four times higher in *H. hermathena* than in the more widely distributed *H. erato* or *H. melpomene* (Fig. 4; Tables S9-S10, Additional file 1).

**Fig. 5:**
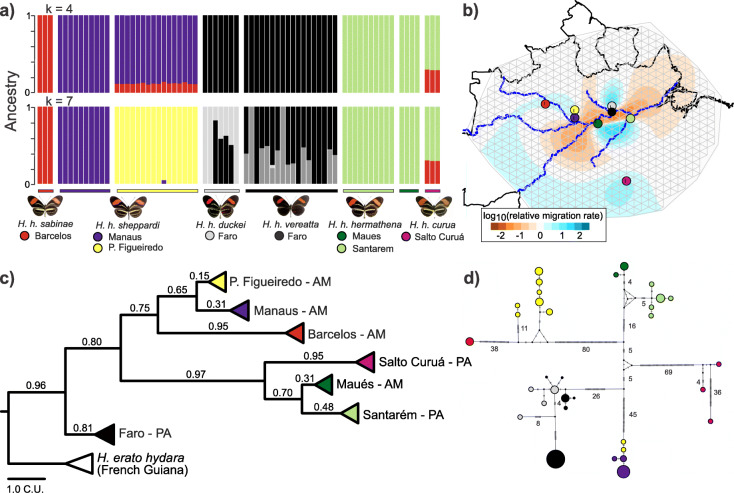
Population structure and gene flow between *Heliconius hermathena* populations. **a** Admixture analysis results for the most likely *k* (4) and the actual number of populations sampled (7). All *k* values tested are shown in Fig. S13 (Additional file 2). **b** Relative migration rates across the range of *H. hermathena*, calculated by EEMS. **c** Bayesian concordance tree generated from 10-kb autosomal windows. Note that both *H. h. vereatta* (gray) and *H. h. duckei* (black) are included in the Faro clade, see Fig. S15 for the full tree (Additional file 2). **d** Mitochondrial DNA haplotype network constructed using whole mtDNA sequences and popArt. Each hash indicates one mutation, and numbers beside branches indicate total numbers of mutations on that branch. The size of each circle is proportional to the number of individuals sharing that mtDNA haplotype. Circles are colored according to the source population

We next inferred *H. hermathena* population structure using *Admixture* and a series of expected numbers of populations (*k* = 2 to *k =* 10; Fig. 5; Figure S13, Additional file 2) [53]. Figure 5a shows the results for the number of populations with the lowest to cross-validation error (4) and the number of localities/subspecies we sampled (7). Most individuals were well-differentiated by geographical location (Fig. 5), particularly populations separated by the Amazon River. The most admixed individuals are found in the Faro population, consisting of *H. h. duckei* and the mimetic *H. h. vereatta.* This strong population structure was also apparent from a haplotype network constructed from whole mtDNA sequences from these 71 individuals, with the exception of two *H. h. sheppardi* individuals from Presidente Figueiredo (hher39, hher40) that grouped with *H. h. sheppardi* from Manaus (Fig. 5; Tables S1 and S11). These results are similar to those based on a mtDNA barcode [27].

The fact that there exist few admixed individuals in any of the core populations suggests that migration is rare between *H. hermathena* populations. Brown and Benson [25] noted that *H. hermathena* do not disperse more than a few hundred meters from their home ranges, probably due to their habitat fidelity and preference for *campina* and *campinarana* over deep forest. This finding further supports the existence of strong barriers to gene flow between geographically close *H. hermathena* populations (Figs. 4 and 5). We next sought to visualize those barriers to gene flow. We estimated effective migration rates between *H. hermathena* populations using EEMS [54]. In contrast to analyses of pairwise *F*_ST_, EEMS estimates migration rates across a geographic region using the geographic locations of and genetic similarity estimates between all populations (Fig. 5b; Figure S14 [54];). Consistent with *Admixture* results, the Amazon River and its tributaries appear to provide strong isolating barriers between the northern (*H. h. sabinae*, *H. h. sheppardi*, *H. h. duckei*, and *H. h. vereatta*) and southern (*H. h. hermathena* and *H. h. curua*) populations: the narrow strip of extremely low effective migration rates matched the course of the Amazon despite the fact that EEMS is agnostic to topography. The only pairs of populations predicted to frequently share migrants were *H. h. duckei* and *H. h. vereatta* from Faro, which are known to frequently hybridize [25] (RRR, personal observation), and the *H. h. hermathena* populations from Santarém and Maués, which were indistinguishable in *Admixture* analyses (Fig. 5a; Figure S13, Additional file 2).

Finally, we reconstructed the phylogenetic relationships among *H. hermathena* populations to begin to understand how *H. hermathena* originated and became so widespread. We constructed a Bayesian concordance tree using autosomal 10-kb windows (Fig. 5c). Similar to ASTRAL quartet scores, concordance factors (CFs) provide an estimate of the congruence between tree topologies estimated from different genomic windows; low CFs may be caused by a variety of factors, including incomplete lineage sorting and gene flow between populations [55]. The concordance tree shown in Fig. 5c suggests that the subspecies groupings are well-supported by whole-genome data (CFs > 0.65), but that geographically adjacent populations are more weakly differentiated (see Figure S15, Additional file 2, for all individuals). Altogether, we find that *H. hermathena* is fragmented into small discrete populations that appear to rarely exchange migrants. This reconstruction also placed the Faro population as the most basal *H. hermathena* clade (Fig. 5c).

### No genome-wide evidence that *H. hermathena* is a hybrid species

We were next interested in determining the origin of *H. hermathena*. *Heliconius hermathena* displays a unique combination of red and yellow color patterns that led several authors to posit that it may have been formed by hybridization between *H. erato* and *H. charithonia* [25, 31, 32]*.* In particular, only *H. hermathena* and the distantly related *H. charithonia* display characteristic rows of submarginal yellow hindwing spots (Fig. 1a). A single putative *H. charithonia* x *H. erato* hybrid has been discovered [56], so it is possible that historical hybridization could have generated a new, distinct species. We tested if *H. hermathena* was formed by hybridization between these *H. erato* and *H. charithonia* by calculating the *D* statistic in 10-kb windows across the autosomes using the species tree (((*H. erato*, non-mimetic *H. hermathena*), *H. charithonia*), *H. melpomene*) [57, 58]. Significantly positive *D* would indicate that *H. hermathena* shares more derived alleles with *H. charithonia* than expected due to incomplete lineage sorting since the three focal species diverged, and therefore a hybrid origin of *H. hermathena*. We found *D* = − 0.015 ± 0.008, suggesting that this is not the case (Table S11, Additional file 1, comparison 5). The Z chromosome exhibited similar values (*D* = − 0.027 ± 0.034; Table S11, Additional file 1, comparison 5). Furthermore, no 10-kb window trees from our phylogenetic analyses grouped *H. hermathena* and *H. charithonia*, and only 24 of 21,247 autosomal trees (0.11%) and no Z trees grouped *H. charithonia* with *H. erato*, *H. himera*, and *H. hermathena*. The proportion of autosomal trees grouping these four taxa was similar to the proportion of trees grouping *H. sara* (0.09%) or *H. demeter* (0.08%) with *H. erato*, *H. himera*, and *H. hermathena*. We therefore found no genome-wide evidence that *H. hermathena* was formed by hybridization between *H. charithonia* and *H. erato*.

### Mimetic *H. hermathena vereatta* originated via introgression from *H. erato*

**Fig. 6:**
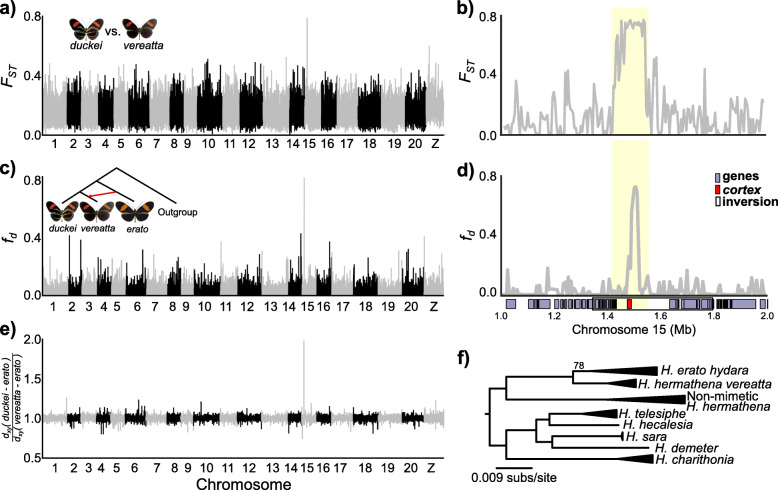
Evolution of the mimetic *Heliconius hermathena* color pattern. **a** Genome-wide *F*_ST_ between mimetic *H. h. vereatta* and non-mimetic *H. h. duckei*, calculated in non-overlapping 10-kb windows. **b***F*_ST_ between *H. h. vereatta* and *H. h. duckei* at the chromosome 15 peak, calculated in 5-kb windows (500-bp step). **c** Genome-wide *f*_*d*_ tests for introgression from *H. erato hydara* into *H. h. vereatta*, calculated in non-overlapping 10-kb windows. *H. melpomene* was used as an outgroup. **d***f*_*d*_ at the chromosome 15 peak. The coordinates of *erato-sara* clade inversion are also shown (see the “Results” and “Discussion” sections and Fig. S17). **e** Relative divergence between *H. h. duckei* and *H. h. vereatta* to *H. erato*. **a**, **b** Calculated using SNP calls relative to the *H. hermathena* reference genome while **c**–**e** calculated using SNP calls relative to the *H. melpomene* reference genome to reduce reference bias. For consistency, all results are plotted relative to the *H. melpomene* reference genome, and all coordinates and gene models are based on the *H. melpomene* genome (the “Materials and methods” section). **f** Maximum likelihood tree based on the variation in the *cortex* region with high *F*_ST_ between mimetic and non-mimetic *H. hermathena* (highlighted yellow in **b** and **d**). Collapsed clades contain multiple individuals of the same species/subspecies. Only branches with less than 100% bootstrap support are labeled. *H. erato hydara* image: Field Museum of Natural History 124251, CC-BY-NC

*Heliconius hermathena* is thought to be one of the rare examples of a non-mimetic *Heliconius* species because its color pattern does not resemble the color pattern of any other co-occurring species [25, 27, 59]. The exception is *H. hermathena vereatta*, which lacks yellow patterns and mimics co-occurring *H. erato hydara* and *H. melpomene melpomene* near the town of Faro [25]. The mimetic color pattern evolved and is maintained in Faro despite frequent interbreeding between *H. h. duckei* and *H. h. vereatta*, suggesting that strong natural selection for mimicry preserves the mimetic form (Fig. 1). We therefore searched for loci associated with yellow presence/absence by scanning for genome regions with high allele frequency differences between *vereatta* and *duckei*. We found a single narrow peak of *F*_ST_ on chromosome 15 containing the known color patterning gene *cortex*. This region showed four-fold higher differentiation than the genome-wide average (Fig. 6). *Cortex* is part of the *H. erato Cr* (*H. melpomene Yb*) locus that controls the presence of the yellow hindwing band across *Heliconius*, and *cortex* expression patterns in pupal wing discs prefigure adult melanic patterns [10]. *Heliconius h. duckei* contained significantly lower *π* (i.e., higher homozygosity) in the *F*_ST_ peak relative to *H. h. vereatta* (0.0009 vs. 0.0057), suggesting that the *vereatta cortex* allele is dominant to the *duckei* allele and melanizes the yellow patterns typical of the other *H. hermathena* subspecies.

*Heliconius erato* populations have evolved similar color patterns by sharing alleles via hybridization distribution, which mediates mimicry between local *H. erato* and *H. melpomene* races [12, 60]. This same process operates in *H. melpomene*, but mimicry between *melpomene*-silvaniform clade species has also frequently been mediated by introgression of color patterning alleles between co-mimetic species [15, 18, 61–63]. We next tested whether *H. h. vereatta* and *H. erato* converged on their shared color pattern independently or via introgression (Fig. 6d). We specifically tested whether *H. hermathena* received its melanic *cortex* allele from sympatric *H. erato hydara* using the *f*_*d*_ statistic (Fig. 6d) [64]. The patterns of *f*_*d*_ mirrored the patterns of *F*_ST_ between *H. h. duckei* and *H. h. vereatta*: we found a single narrow peak of *f*_*d*_ just upstream of *cortex*, suggesting that the *H. h. vereatta* color pattern was formed by introgression of a *cortex* allele from *H. erato* into a *H. h. duckei*-like ancestor (Fig. 6c, d). Consistent with this hypothesis, divergence (*d*_*xy*_) between *H. h. vereatta* and *H. erato* was significantly lower in this region than genome-wide or between *H. h. duckei* and *H. erato*, and ML analysis of variation in this region grouped *H. erato* and *H. h. vereatta* (Fig. 6e, f).

While we detected no genome-wide evidence for hybridization between *H. hermathena* and *H. charithonia*, it is also possible that these species share color patterns because they shared color patterning alleles via hybridization. Alternatively, hybridization may be so ancient that its signal has been eroded by continued divergence. However, we found no *f*_*d*_ signatures suggesting that *cortex* alleles have been shared between *H. hermathena* and *H. charithonia* (Figure S16). Interestingly, the ML tree in Fig. 6f showed that *H. charithonia* was placed outside a clade containing its sister species *H. demeter* and *H. sara*, and *H. telesiphe* and *H. hecalesia*. Edelman et al. [20] showed that there was at least one ancient hybridization event between the ancestors of *H. sara*/*H. demeter* and *H. hecalesia*/*H. telesiphe* that transferred a large (~ 500 kb) inversion centered on *cortex*. The tree in Fig. 6f suggests that *H. charithonia* carries the ancestral standard allele in this region, and, indeed, we found (1) two large *H. charithonia* scaffolds (126 kb and 263 kb) that span the inversion breakpoints in the standard orientation and (2) increased *d*_*xy*_ between *H. charithonia* and species carrying the inversion in this region (Figure S17, Additional file 2). Thus, *H. erato*, *H. himera*, *H. hermathena*, and *H. charithonia* share ancestral non-inverted haplotypes in the *cortex* region, perhaps suggesting that *H. hermathena* and *H. charithonia* yellow patterning alleles arose independently or are controlled by ancestral variation in this region. The mimetic *H. h. vereatta* color pattern then appears to be a derived phenotype mediated by introgression of a small upstream region of *cortex* from co-occurring *H. erato.*

## Discussion

Over 150 years of *Heliconius* research has provided many key insights into the genetic basis of color patterns, the evolution of Müllerian mimicry, and the fluidity of species boundaries [3, 4, 20, 56, 65]. However, detailed investigations of unique populations continue to provide insight into adaptation, speciation, and the genomic patterns that these processes leave behind. Here, we used whole-genome sequencing data to test hypotheses about the effects of population structure and hybridization on the genetic health and color pattern evolution of two rare *Heliconius* species.

### Influence of population fragmentation and recent bottlenecks on the genetic health of *H. nattereri* and *H. hermathena*

Population fragmentation and reduced gene flow can severely reduce genetic diversity within species [66]. The distributions of white sand habitats and Atlantic Forests have significantly shrunk since the end of the last glacial maximum ~ 12,000 years ago, fragmenting populations of species that inhabit them. This habitat loss and fragmentation have only increased over the last century as logging and climate change have rapidly taken their toll [67–70]. It is therefore critical to understand how historical fragmentation and habitat loss have affected the current genetic health of the organisms that inhabit these threatened environments and to use those data to estimate the vulnerability of those species to contemporary habitat loss.

*Heliconius nattereri* has been found in only four areas of Atlantic Forest that are each surrounded by large areas of unsuitable habitat and deforested areas, leading to the current patchy distribution of this threatened species [21–23, 71]. While local population densities can be high, most collection efforts have produced few specimens from any of these four sites. Interestingly, *H. nattereri* carries a perhaps surprising amount of genetic diversity (*π*) considering its small census sizes: 36% of the diversity found in its widespread relative *H. melpomene* and about the same as the cosmopolitan fly *Drosophila melanogaster* [72]. However, much of the genetic variation in *H. nattereri* appears to be deleterious (Fig. 3). Furthermore, mean Tajima’s *D*, the high variance of *D*, and historical population size estimates suggest that *H. nattereri* experienced a recent population bottleneck (Fig. 2). Combined with the continued loss of Atlantic Forest habitats through climate change and deforestation [70], limited host plant ranges and extended larval and pupal development times relative to their competitors, it seems likely that Brown [22] (p. 53) was correct when he wrote that “*H. nattereri* seems to be a very primitive, inflexible, sensitive, and evidently declining species, with a rather uncertain future.”

In contrast to *H. nattereri*, *H. hermathena* is widespread across the Amazon River basin but inhabits unique white sand ecosystems [25–27]. We found that *H. hermathena* population size and deleterious mutation load estimates are surprisingly similar to those in *H. nattereri* (Figs. 2 and 3), suggesting that their patchy distribution, strong isolation between patches, and recent population bottleneck have significantly reduced their genetic health. Brown and Benson [25] suggested that *H. hermathena* was once widespread and then fragmented into isolated populations due to forest growth after the last glacial maximum, ~ 12,000 years ago. Alternatively, *H. hermathena* may have originated in a single patch and then dispersed to other patches. While our data do not allow us to definitively distinguish between these two scenarios, they suggest the former scenario. *Heliconius hermathena* population sizes began to diverge ~ 10,000 years ago, suggesting that they were part of a single population until that point (Fig. 2), but those populations are strongly genetically differentiated today (Fig. 5). Genetic diversity is about twice as high in populations north of the Amazon, with the center of diversity near Manaus and Presidente Figueiredo (Figure S14, Additional file 2), yet all populations have similarly low levels of *π* and Tajima’s *D* signatures of recent bottlenecks (Fig. 2). If *H. hermathena* recently spread from a single location, we may expect *π* to decrease with increasing distance from the source population. Phylogenetic reconstructions suggested that the Faro population is an outgroup to the remainder of the *H. hermathena* populations (Fig. 5c; Figure S15, Additional file 2).

Low migration rates among populations are likely due to this species’ habitat fidelity and generally low dispersal (a few hundred meters [25];). Dispersal between patches does appear to occur at low rates, though as a single *H. h. sheppardi* individual from Presidente Figueiredo appears to be slightly admixed with both *H. h. sheppardi* from Manaus and *H. h. vereatta* from Faro (according to Fig. 5; Figure S13, Additional file 2). Furthermore, red ventral hindwing spots characteristic of the Faro populations are occasionally found in *H. h. hermathena* on the southern bank of the Amazon [25]. Overall, the strong population structure we observe in *H. hermathena* has likely led to the current low effective population sizes and significant deleterious mutation loads (Figs. 4 and 5).

### Evolution of non-mimetic *H. hermathena*

Hybridization between closely related species allows gene flow that may transfer beneficial alleles between species or even produce entirely new species [32, 73]. *Heliconius* butterflies have provided key insights into the role of hybridization in creating and blurring species boundaries [15, 16, 20, 29]. At least three species (*H. heurippa* [29], *H. hecalesia* [20, 37], and *H. hortense/H. clysonymus* [19]) appear to be hybrid species. Importantly, evidence for the hybrid origin of *H. hortense* and the ancient hybridizations in the *erato-sara* clade were only uncovered with whole transcriptome and whole genome data, respectively, highlighting the importance of using genome-wide data to test hypotheses about hybridization. Despite speculation based on their unique color pattern combination and discordant placements in analyses of mitochondrial and nuclear markers [30–32], we found no evidence from whole-genome alignments or polymorphism data that *H. hermathena* was formed by hybridization between *H. charithonia* and *H. erato*. Our results thus reinforce the need to test for introgression with genomic data.

The yellow color patterns of *H. hermathena* and *H. charithonia* are therefore controlled by (1) variation evolved independently in the two lineages, (2) shared ancestral variation, or (3) alleles shared via small-scale introgression. We found little evidence for introgression between *H. hermathena* and *H. charithonia*, especially near known color patterning genes, including *cortex* (Figure S11, Additional file 2). The *cortex* region itself contains multiple color patterning genes, as evidenced by the number of genetically separable color patterning loci in this region (e.g., *H. erato Cr*, *H. melpomene Yb*, *Sb*, and *N*) and the complex color pattern switches that it controls in *H. numata* [65, 74]. The fact that *H. charithonia* and *H. hermathena* carry the ancestral, non-inverted allele might suggest that their yellow patterns are controlled by ancestral variation (Fig. 6f). In any case, these findings raise the further question of why this color pattern has been maintained over such a long period of time, as these two species diverged ~ 13 million years ago [36]. Non-mimetic *H. hermathena* primarily inhabit shady undergrowth and fly erratically between shadowy patches, leading Brown and Benson [25] to suggest that the color pattern and flash-disruptive flight behavior that defies “visual following for any distance … would probably be a near optimal means of avoiding predation.” The effectiveness of the aposematic, but non-mimetic color pattern seems to be supported by the narrow distribution of mimetic *H. h. vereatta* in the southern Faro population, the only location where *H. hermathena* overlaps the distributions of its co-mimics *H. erato hydara* and *H. melpomene melpomene*. On a finer scale, mimetic *H. h. vereatta* prefers more open habitats on the edges of fields where its co-mimics also fly [25] (RRR, personal observation).

### Müllerian mimicry mediated by adaptive introgression of a color pattering allele in the *erato-sara* clade

The evolution of the mimetic *H. hermathena* color pattern provides a unique example of phenotypic convergence between co-occurring species via sharing of color pattern alleles through small-scale introgression. Numerous examples of this process have been documented in the *melpomene*-silvaniform clade, including sharing of *optix* alleles between *H. melpomene*, *H. timareta*, and *H. elevatus* [11, 15, 61]; introgression of *H. melpomene optix* alleles to the silvaniform *H. besckei* [18]; introgression of *aristaless1* alleles from *H. cydno* to *H. melpomene* [13]; introgression of a *P* supergene allele from *H. pardalinus* to *H. numata* [63]; and introgression of *cortex* alleles between *H. melpomene* and *H. cydno* [62]. However, this process has only recently been documented in the *erato-sara* clade. Van Belleghem et al. [12] and Lewis et al. [60] found evidence that *H. erato* races converged on similar color patterns via sharing of alleles of these same color patterning genes among populations, while Edelman et al. [20] found evidence for ancient introgression of a large inversion, centered on *cortex*, between the ancestors of *H. sara/H. demeter* and *H. telesiphe/H. demeter. H. hermathena* therefore provides a unique example of recent adaptive introgression of a small color patterning allele between two distinct *erato-sara* clade species. Our analyses suggest that an ancestral, non-mimetic *H. h. duckei*-like population hybridized with *H. erato* and that selection for mimicry maintained the *H. erato cortex* allele in southern Faro despite continued backcrossing of the novel mimetic *H. hermathena* with the non-mimetic ancestor (Fig. 6). The narrow interval of high *F*_ST_, *f*_*d*_, and relative *d*_*xy*_ between *H. h. duckei* and *H. h. vereatta* suggests that selection for mimicry is very strong in the southern Faro population and/or that hybridization occurred a long time ago. Divergence between the *H. erato* and *H. h. vereatta cortex* alleles is 0.0091 relative to the genome-wide average of 0.0153, providing a rough estimate that the introgression occurred ~ 1.5 million years ago (~ 60% of the total time since *H. hermathena* and *H. erato* diverged), although divergence in the introgressed region may have been affected by other factors such as natural selection, recombination, or different mutation rates.

Finally, many different loci epistatically interact to produce the final wing color pattern (e.g., [60]). *H. h. vereatta* and *H. h. duckei* forewing bands are noticeably redder than other *H. hermathena* populations, where the bands are more orange (Fig. 1). This could be due to the additional sequence evolution at other color patterning loci, or it is even possible that the *H. hermathena*/*H. erato* hybridization event introduced other, small-effect variants and loci other than *cortex* that produce these more subtle differences between the Faro subspecies and the remainder of *H. hermathena* populations. However, we do not find strong differentiation at the other major color patterning genes. The broad hump of slightly higher *F*_ST_ on chromosome 10 is centered ~ 3 Mb to the right of *WntA* and is a collection of widely scattered windows. The 10-kb window containing *WntA* has *F*_ST_ of 0 (48 SNPs). The window with the highest *F*_ST_ on chromosome 10 (0.28, 104 SNPs) is 370 kb away from *WntA*. In contrast, the 10-kb windows containing *cortex* all have *F*_ST_ > 0.70 (> 62 SNPs).

### Phylogenetic placement of *Heliconius nattereri*

Recent species tree reconstructions based on tens to thousands of gene trees have consistently found widespread discordance in the *melpomene*-silvaniform clade, suggesting that these species, especially the silvaniforms, frequently hybridize with each other [20, 36]. We found the same patterns of discordance among gene trees and between datasets after including our high-quality *H. nattereri* genome in these analyses, showing that the relationships in this clade remain unresolved [20]. Interestingly, the *H. nattereri* genome has also been shaped by the rampant gene flow among silvaniforms, which is reflected by the different placements of *H. nattereri* in species trees based on different datasets: summaries of autosomal gene trees placed *H. nattereri* as an outgroup to the remaining *melpomene*-silvaniform clade species (Fig. 1; Figure S7-S8, Additional file 2); Z-linked gene trees placed a clade of *H. nattereri*, *H. numata*, and *H. besckei* as an outgroup to the remaining species (Figure S9), while mtDNA places *H. nattereri* in the middle of a monophyletic silvaniform clade (Fig. 1). The addition of the *H. nattereri*, *H. hecale*, and *H. elevatus* genomes to the Edelman et al. [20] dataset therefore does little to resolve relationships within this intriguing clade. The Z exhibits generally greater differentiation and more resistance to gene flow than the autosomes, and analyses of the Z can provide better supported gene trees [16, 17, 37]. These observations suggest that, at the least, *H. nattereri* hybridizes much less frequently with the remaining silvaniforms than the other silvaniforms hybridize with each other. Hybridization within the silvaniforms affects *H. nattereri*, but perhaps less than the remaining silvaniforms. The mtDNA relationships may be due to mitochondrial capture from one of these hybridization events.

## Conclusions

*Heliconius* butterflies have been studied for over 150 years because of their bold, aposematic color patterns that frequently mediate mimicry among distantly related species. Recent genomics studies of the widespread species *H. melpomene* and *H. erato* have provided exciting insights into the important roles that hybridization and introgression play in speciation and color pattern evolution. Our results provide a striking contrast to the patterns that have emerged from years of study focused on abundant and widespread species such as *H. erato*, *H. melpomene*, and their close relatives. We showed that both *H. nattereri* and *H. hermathena* have substantially reduced genetic diversity and high deleterious mutation loads relative to other *Heliconius*. These are the result of low effective population sizes, recent population bottlenecks, and pronounced population subdivision. Despite recurrent speculation based on its suggestive color pattern combination, we detect no genomic evidence that *H. hermathena* is a hybrid species. However, we do find that the one mimetic subspecies of *H. hermathena* acquired its mimetic phenotype via hybridization with *H. erato*. We thus provide a unique example of mimicry evolution caused by recent adaptive introgression across species boundaries. Our results further support the conclusion that this process is a general feature of *Heliconius* color pattern evolution when viewed with other recent analyses of intraspecific and ancient hybridization in the *erato-sara* clade, and well-documented introgression in the *melpomene*-silvaniform clade.

## Materials and methods

### Genome assemblies of *H. hermathena* and *H. nattereri*

We isolated DNA from the thorax of four wild-caught *H. hermathena* adult females, then pooled all four DNA samples before constructing sequencing libraries. DNA was pooled to produce a uniform sample with enough DNA to construct all paired-end and mate-pair libraries, which required 14 μg. We constructed Illumina paired-end (PE) libraries with insert sizes of 250 and 500 bp using the KAPA Hyper Prep Kit (KR0961 – v1.14) and 1 μg genomic DNA. We constructed mate-pair (MP) libraries with insert sizes of 2 kb, 6 kb, and 15 kb using the Nextera Mate Pair Library Prep kit (FC-132-1001) and 4 μg genomic DNA. We then pooled these libraries in a ratio of 50:18:10:17:4 and sequenced them 2 × 100 bp on a single lane of Illumina HiSeq 4000 (Table S2, Additional file 1). We trimmed low-quality regions and remaining adapters from raw PE reads using Trimmomatic v0.36 [75] and from MP libraries using the platanus_internal_trim tool from Platanus v1.2.4 [76]. Trimmed libraries were assembled and scaffolded using Platanus v1.2.4 (default settings) and the assembly polished using Redundans v0.13a (default settings) [77]. We removed scaffolds < 5 kb from the assembly, then generated species-specific repeat libraries and masked repeats using RepeatScout v1.0.5 and RepeatMasker v4.0.8, respectively, to produce the final *H. hermathena* reference genome assembly [78, 79]. We sequenced and assembled the *H. nattereri* genome using an identical protocol with genomic DNA extracted from thoraxes of two wild-caught males and two females (Tables S1-S2, Additional file 1). We calculated the frequency spectrum of canonical 21-mers in raw reads from the PE 250 bp libraries using jellyfish 2.2.3 [80], then input the resulting histograms into GenomeScope to estimate genome size, heterozygosity, and sequencing error rates [34].

In addition, we assembled a draft *H. charithonia* genome using publicly available data for *H. charithonia charithonia* from Puerto Rico (accessions SRR4032025 and SRR4032026) and Platanus version 2.0.2 [81]. We trimmed adapter sequences using TrimGalore v0.6.1 (http://www.bioinformatics.babraham.ac.uk/projects/trim_galore/) and cutadapt v1.18 [82], then identified and removed overrepresented sequences using FastQC v0.11.5 (https://www.bioinformatics.babraham.ac.uk/projects/fastqc/) and RemoveFastqcOverrepSequenceReads.py (last accessed 2019-11-25; available https://github.com/harvardinformatics/TranscriptomeAssemblyTools). GenomeScope analyses showed that SRR4032025 exhibited low heterozygosity (0.67%) and error rates (0.12%) relative to SRR4032026 (3% and 0.41%). The estimated genome size for *H. charithonia* from SRR4032025 was 303 Mb, similar to its close relatives (Table 1). We therefore assembled contigs using Platanus v2.0.2 and the processed reads from SRR4032025, then scaffolded and phased contigs using reads from both accessions. Finally, we removed scaffolds < 5 kb and identified and masked species-specific repeats using the pipeline outlined above.

We assessed the quality of our assemblies and other well-assembled nymphalid genomes using BUSCO v3 and the endopterygota gene set (2440 single-copy orthologs) from OrthoDB v9 [35, 83]. The accessions of the genome assemblies that we tested are as follows: *B. anynana* (RefSeq GCF_900239965.1), *D. plexippus* (GenBank GCA_000235995.2), *H. erato demophoon* v1 (www.lepbase.org [LepBase] v4), *H. e. lativitta* v1 (LepBase v4), *H. melpomene* v2.5 (LepBase v4), *Hypolimnas misippus* (GenBank GCA_008963455.1), *J. coenia* v1 (LepBase v4), *L. arthemis* (BioProject PRJNA556447), *M. cinxia* (GenBank GCA_000716385.1), and *V. tameamea* (GenBank GCA_002938995.1).

Finally, we assigned *H. hermathena* and *H. nattereri* scaffolds to the *H. erato demophoon* v1 and *H. melpomene* v2.5 assemblies, respectively, using RaGOO [84]. We used this chromosome assignment information in plotting chromosome-level plots and to identify sex-linked scaffolds.

### Whole-genome alignments and genome-wide consensus phylogeny

We added our new *H. nattereri*, *H. hermathena*, and *H. charithonia* reference genomes to the whole-genome alignment generated by Edelman et al. [20] using the progressiveCactus aligner (commit 348815c from September 19, 2019) [38, 39]. We downloaded the Edelman alignment from Dryad (10.5061/dryad.b7bj832) and extracted all sequences using hal2fasta. We made three modifications to the Edelman alignment based on the mtDNA phylogeny and previous publications (see Figure S1, Additional file 2). First, we added *H. hermathena* to the branch leading from Anc17 to Anc21 to yield the subclade (((*H. erato*, *H. himera*), *H. hermathena*), *H. hecalesia*). Next, we added *H. charithonia* as sister to *H. sara* to yield the new subclade ((*H. charithonia*, *H. sara*), *H. demeter*). Finally, we re-aligned the silvaniform clade using a polytomy, including *H. elevatus* and *H. hecale* (available from LepBase v4) and *H. nattereri*. These guide trees simply aid the alignment of the sequences and do not greatly affect downstream analyses if the species are closely related [20].

We followed Edelman et al. [20] to infer species trees, using all 18 *Heliconius* species, as well as just the eight species from the *erato* clade or 10 species from the *melpomene* to improve and/or confirm resolution of relationships in those clades. We included *Eueides tales* in the full *Heliconius* dataset, *H. melpomene* in the *erato* dataset, and *H. erato* in the *melpomene* dataset as outgroups. We used the *H. melpomene*, *H. erato*, and *H. melpomene* genomes as reference genomes for these three datasets, respectively, when extracting alignments using the HAL toolkit.

Extensive gene flow among *Heliconius* species has resulted in a mosaic of gene genealogies across the genome. Edelman et al. [20] inferred gene trees for non-recombining regions, then summarized these gene trees using ASTRAL, which analyzes four-taxon groupings to provide support for each branch in the inferred species phylogeny. ASTRAL and other species tree inference methods assume that each gene tree reflects a single topology, an assumption that is violated if historical recombination has occurred within the gene or window being analyzed. To minimize the number of violations, we inferred phylogenies for small windows with weak evidence for recombination, then summarized them using ASTRAL-iii, similar to Edelman et al. [20]. Linkage disequilibrium is essentially gone within ~ 10 kb in *H. melpomene*.

For each of the three datasets (all *Heliconius*, *erato* clade, and *melpomene* clade), we extracted alignment blocks from all 10-kb windows from the reference genome, then filtered alignment blocks for single-copy coverage in all species (therefore excluding ambiguously aligned regions and taxon-specific duplications). We stripped all sites with a gap in any species and tested for recombination within each alignment using PhiPack [85], which uses patterns of variation to infer historical recombination events within an alignment. PhiPack reports the probability of NO recombination within the alignment; smaller *p* values suggest that there is stronger evidence for recombination within the window. We used all autosomal alignments containing sequences for all focal species, at least 1000 aligned bp, at least 100 phylogeny-informative sites, and a probability of recombination *p* > 1e−10. We chose these cutoffs to include a reasonable number of high-quality windows for each comparison. Figures S2 - S6 (Additional file 2) contain more detailed information on each dataset’s characteristics prior to filtering. These filters produced 8674 valid alignments for the *Heliconius* dataset, 22,430 windows for the *erato* clade, and 3601 windows for the *melpomene* clade. We inferred trees for each valid window using IQtree after selecting the best model using ModelFinder and estimated statistical support using 1000 ultrafast bootstraps. Finally, we inferred the species tree for each dataset using ASTRAL-iii using its exact algorithm [41].

The primary source of the discrepancy in the number of windows is the filter for evidence of recombination within the window, especially in the *melpomene* clade (Figure S5). We therefore repeated the *Heliconius* dataset analysis following Edelman et al. [20] using fully aligned blocks from coding and non-coding regions, which tend to be short and therefore have little or no evidence for recombination (Figure S3, S4, and S7, Additional file 2). We constructed trees using all autosomal alignments with at least 150 bp, at least 10 informative sites, and probability of recombination > 1e−4. The final coding and non-coding datasets consisted of 12,369 and 29,071 alignments, respectively. The ASTRAL trees are consistent with the 10-kb window trees and are presented in Figure S7 (Additional file 2).

Finally, we tested consistency between the ASTRAL method and BUCKy using the filtered alignments from the *erato* and *melpomene* clade datasets (Figure S9, Additional file 2). We inferred gene trees using MrBayes 3.2 under a GTR model with gamma-distributed rates with two runs of 1000,000 generations and then summarized the results using BUCKy 1.4.4 [86].

### *Heliconius* mitochondrial genome assemblies

We downloaded 31 *Heliconius* species samples, including seven complete mitochondrial genomes, from NCBI (Table S3, Additional file 1). We used NOVOplasty to extract and assemble mtDNA reads from these accessions using bait sequences indicated in Table S3, a *k*-mer size of 39, and an expected size range of 12–22 kb [43]. We aligned all mtDNA genomes using the multiple circular sequence alignment program MARS [87]. Finally, we inferred the mtDNA phylogeny using IQtree v1.6.12 using the best-fit model determined using ModelTest and estimated support using 1000 ultrafast bootstraps [42, 88, 89].

### Whole-genome resequencing

We collected adult *H. hermathena* specimens from the states of Amazonas and Pará in northern Brazil between 2010 and 2017 (Fig. 1; Table S1, Additional file 1). We collected adult *H. nattereri* from the states of Espírito Santo and Bahia in 2015 and 2017. We extracted genomic DNA from thorax tissues from each of these butterflies using a phenol-chloroform DNA extraction protocol, then constructed paired-end libraries using a KAPA Hyper Prep Kit (KAPA Biosystems) and sequenced them to ~ 20X coverage using 2 × 100 bp Illumina HiSeq 2500 (Table S1, Additional file 1). Note that we also re-sequenced *H. nattereri* individuals that we used for genome assembly because (1) these samples were extremely rare and (2) we had to pool DNA extractions to construct the libraries for genome sequencing and assembly. We did not re-sequence *H. hermathena* samples used for genome assembly because we had many additional samples (Table S1, Additional file 1).

### SNP calling pipeline

We used five different SNP call sets for our analyses: (1) *H. nattereri* samples relative to the *H. nattereri* reference genome, (2) *H. hermathena* samples relative to the *H. hermathena* reference genome, (3) *melpomene*-silvaniform clade samples relative to the *H. melpomene* v2.5 reference genome, (4) *erato-sara* clade samples relative to the *H. erato demophoon* v1 reference genome, and (5) *erato*-*sara* clade samples relative to the *H. melpomene* v2.5 reference genome. All SNP call sets were generated using the same pipeline. We trimmed adapters and low-quality regions from raw re-sequencing reads using TrimGalore 0.6.1 and cutadapt v1.18 [82], then removed reads containing overrepresented sequences (identified using FastQC). We then mapped reads to the appropriate reference genome using Bowtie2 v2.3.0-beta7 with the parameter “--very-sensitive-local” [90]. We marked PCR duplicate reads using PicardTools v2.8.1 and realigned around indels using the Genome Analysis ToolKit’s (GATK, v3.8) RealignerTargetCreator and IndelRealigner. Finally, we called SNPs using the GATK UnifiedGenotyper with default settings except the heterozygosity prior was set to 0.02, minimum allowable base quality scores were set to 30, and minimum mapping quality set to 20 [91].

### *SMC++* analyses

We inferred changes in ancestral population sizes using the sequentially Markovian coalescent implemented in *SMC++* v1.15.3 [47]. We used *H. hermathena* SNP calls relative to the *H. hermathena* reference genome and *H. nattereri* SNP calls relative to the *H. nattereri* genome for these analyses. We filtered out genotypes with quality < 10, then removed SNP sites with any missing genotypes or multiple alternate alleles. We also excluded sex-linked sites from this analysis. We estimated historical population sizes using the *SMC++ estimate* function, using all samples as distinguished lineages, setting the thinning parameter *k* = 1000 × ln (no. of samples in analysis × 2), using a mutation rate of 2.9e−9 per bp per generation [45, 47] and assuming four generations per year. We estimated the split time of the northern and southern *H. nattereri* populations using the *SMC++ split* function. Previous studies of historical *Heliconius* population sizes used PSMC (e.g., [17]). We provide PSMC results for *H. nattereri* and a sample of *H. hermathena* in Figure S12 (Additional file 2) for comparison.

### *H. nattereri* and *H. hermathena* mutation load analysis

We used SNP calls from *melpomene*-silvaniform clade samples aligned to the *H. melpomene* v2.5 reference genome or *erato*-*sara* clade samples aligned to the *H. erato demophoon v1* reference genome for this analysis. We calculated nucleotide diversity per site (*π*), Tajima’s *D*, and *F*_ST_ in 10-kb non-overlapping windows using VCFtools 0.1.15 [92]. For the *H. nattereri* mutation load analysis, we determined the ancestral state for each site using *H. cydno* and *H. atthis* data, requiring all eight samples called and agreement from a majority of alleles (see Table S4, Additional file 1, for sample information). We annotated the effects and impacts of derived mutations on *H. melpomene* v2.5 gene models using snpEff 4.3T [51], then summarized fixed and polymorphic derived alleles in *H. nattereri*, *H. melpomene melpomene* (French Guiana), *H. m. rosina* (Panama), and *H. pardalinus.* We chose six random Santa Teresa *H. nattereri* samples and six random *H. m. rosina* samples for this analysis to match the number of *H. pardalinus* samples. We considered an allele to be fixed in a population if it was present in all individuals from that population. We considered mutations with the following snpEff annotations as “neutral”: synonymous_variant, intron_variant, and NONE. We considered mutations with the following annotations as “deleterious”: missense_variant, start_lost, and stop_gained. We analyzed a subset of *H. hermathena* populations similarly, including six random individuals from each *H. h. sheppardi* population, *H. h. duckei*, *H. h. vereatta*, and *H. h. hermathena* (Santarém). We analyzed the six French Guiana *H. erato hydara* individuals for comparison. We polarized SNP states using *H. clysonymus*, *H. hortense*, and *H. charithonia* (2 samples). We required all four samples to be called and a majority of alleles to agree in order to assign an ancestral state to a particular SNP site.

### *Heliconius* genetic differentiation by distance

We used SNP calls from *melpomene*-silvaniform clade samples relative to the *H. melpomene* reference genome and *erato-sara* clade samples relative to the *H. erato* reference for this analysis. For each species *H. melpomene*, *H. nattereri*, *H. hermathena*, and *H. erato*, we estimated the weighted, genome-wide average *F*_ST_ [93] between all pairs of sampled populations using VCFtools v0.1.15. Negative *F*_ST_ estimates were set to 0. We then estimated geodesic distances between populations based on latitude and longitude coordinates using the distGeo() function of the geosphere R package. If a population contained three or more individuals, we used the midpoint calculated using a geographic midpoint calculator (http://geomidpoint.com/) as the population location. In these cases, the midpoint of the segment connecting both populations was used for geodesic distance estimation.

### *H. hermathena* population structure

We estimated the ancestry of *H. hermathena* individuals using ADMIXTURE v1.3.0 [53]. We used *H. hermathena* SNP calls relative to the *H. hermathena* reference genome for this analysis. We first filtered all genotypes with quality < 10, then included only sites with no missing genotypes and minor allele frequency greater than 5%. Finally, we performed linkage disequilibrium-based pruning using PLINK 1.90 [89], keeping only variants with *r*^2^ < 0.1 in sliding 50-site windows (10-site step). This resulted in 236,596 sites in the final filtered dataset. The most likely number of clusters was selected based on cross-validation error (CV) and the value of *k* that minimizes the residuals [94]. We ran ADMIXTURE with cross-validation for values of *k* from 2 to 10.

We visualized spatial patterns in genetic diversity and regions of gene flow using the program EEMS (estimated effective migration surfaces) [54]. We used the same SNP set generated for ADMIXTURE for this analysis. We calculated the dissimilarity matrix using the bed2diffs program included in the EEMS package. An outer coordinate file was generated using the add path method in the Google Earth Pro (v. 7.3.2.5776) tool. We tested several deme sizes (300, 450, 750) using the runeems_snps version of eems. We set the number of demes to 750 and defined the outer boundary of the region by the polygon (in latitude-longitude coordinates of the focal populations; Table S1, Additional file 1). This number of demes provided fine resolution. We ran the MCMC across 10 independent 10,000,000-step runs logged every 2000 steps and 2,500,000 set as burn-in. The results were combined across the independent analyses using the reemsplots R package (available with EEMS), and convergence of runs was visually assessed (Figure S18, Additional file 2). Using this package, we plotted the geographic distance and genetic dissimilarity across demes and generated surfaces of effective diversity (*q*; Figure S14, Additional file 2) and effective migration rates (*m*; Fig. 5).

### *H. hermathena* Bayesian concordance tree construction

We aligned reads and called SNPs for each *H. hermathena* sample and three *H. erato hydara* samples (SRR4032061, SRR4032068, and SRR4032074) using the *H. hermathena* reference genome and the pipeline outlined above. We used the GATK’s FastaAlternateReferenceMaker to generate a consensus fasta for each sample, including IUPAC ambiguities at heterozygous sites. We then split the genome into non-overlapping 10-kb windows, picked 5000 random autosomal windows, and estimated trees for each window using MrBayes 3.2 with a GTRγ substitution model and 6 rate categories [95]. Each MrBayes analysis consisted of two runs of 1000,000 generations and samples every 500 generations. We then used BUCKY 1.4.4 to summarize trees from all 5000 runs, discarding the first 25% of trees from each run, see Figure S15 (Additional file 2).

### *H. hermathena* mtDNA haplotype network

We assembled complete mtDNA sequences for all *H. hermathena* samples and constructed a haplotype network to infer population structure (Fig. 5d). We performed quality control of raw Illumina reads using FastQC and MultiQC [96]; adapters were removed using Trim Galore. Genomic data was subsequently filtered for mtDNA reads using the package MIRAbait of the MIRA assembler v. 4.0, using the mitogenome of *Heliconius melpomene rosina* (NCBI Accession Number KP153600) as a reference, and *k*-mer size 15, since higher *k*-mer values resulted in poorer mitogenome assembly. Analyses were run in the Galaxy platform [97] with default parameters for each program (except for *k*-mer value in MIRAbait). We imported all files into Geneious v. 10, where the mitogenome of *Heliconius hermathena* was assembled (using the Map to Reference command, and *H. melpomene rosina* mitogenome as reference), based on the paired reads of all individuals. *H. hermathena* sequencing reads from each individual were then used in a Map to Reference procedure against *H. hermathena* mitogenome to assemble all individual mitogenomes. We then aligned mitogenomes with the MAFFT [98] plugin in Geneious v. 10 using the FFT-NS-i × 1000 algorithm. Protein coding genes (PCGs) were extracted and concatenated using the Concatenate sequences or alignments command in Geneious resulting in the final alignment comprising all individuals; only the PCGs were used in subsequent analyses. Finally, we visually checked the alignment for possible errors with AliView [99].

The final alignment was imported into DnaSP v. 6 [100] to assess *π*. Each individual in the alignment was assigned to their specific subpopulation defined by its sampling locality (Data > Define Domain Set). Overall and intrapopulation *π* values were obtained with the DNA Polymorphism command, first for all subpopulations combined and then for each of the defined subpopulations separately. Population differentiation was measured by overall and pairwise *F*_ST_ using Arlequin v. 3.5 [101] computed for 100 permutations and calculating the distance matrix for pairwise differences. A haplotype network for the mitogenome sequences of all individuals was estimated using the program PopArt v. 1.7 [102], with the median-joining network algorithm (epsilon = 0), see Table S11 (Additional file 1) and Fig. 5d.

### Analysis of *H. hermathena* hybridization and mimicry locus

We searched for evidence of hybridization using the *D* statistic [57, 58]. We processed and aligned reads and called SNPs using data from *H. erato hydara* (French Guiana), *H. hermathena vereatta*, *H. hermathena duckei*, *H. hermathena sheppardi*, *H. hecalesia*, *H. telesiphe*, *H. sara*, *H. demeter*, *H. charithonia*, and *H. melpomene nanna* and the pipeline detailed above, but mapped all reads to the *H. melpomene* v2.5 reference genome assembly to minimize reference bias (Tables S1 and S3, Additional file 1). We randomly sampled six individuals from populations with more than that number. We then calculated *D* in 10-kb non-overlapping autosomal windows using Simon Martin’s genomics_general toolkit available at https://github.com/simonhmartin/genomics_general (commit 3e9281b from September 24, 2019). Values shown in Table S12 (Additional file 1) are the average and standard deviation (calculated using block jackknifing with 95% of the data).

We calculated *F*_ST_ between *H. h. vereatta* and *H. h. duckei* in 10-kb non-overlapping (genome-wide) or 5-kb sliding (500-bp step, focal region) windows using VCFtools 0.1.15. We used variant calls from these subspecies relative to the *H. hermathena* reference assembly. We used the full *D* statistic call set described in the previous paragraph to test if mimetic *H. hermathena* alleles originated via introgression from *H. erato* by calculating the *f*_*d*_ in 10-kb non-overlapping windows (genome-wide) and 5-kb sliding windows (500-bp step; focal region) using the tree (((*H. h. duckei*, *H. h. vereatta*), *H. erato*), *H. melpomene*). Negative *f*_*d*_ values were set to 0 before plotting, as these are meaningless [64]. We used the same *D* statistic callset to calculate *d*_*xy*_ in 10-kb non-overlapping windows using the genomics_general toolkit. To be consistent, we plotted all Fig. 6 results relative to the *H. melpomene* v2.5 reference genome. We assigned *H. hermathena* scaffolds to *H. melpomene* scaffolds using RaGOO [84], then used this information during plotting with the gwplotting R package available at https://github.com/nwvankuren/gwplotting. We also used this package to plot *H. melpomene* gene models.

We reconstructed a maximum likelihood tree for the 100 kb peak of *F*_ST_ between *duckei* and *vereatta* using IQtree and the best model chosen by ModelTest, including 1000 ultrafast bootstraps to estimate support. For this analysis, we extracted alternate FASTAs for each sample using the GATK FastaAlternateReferenceMaker, including IUPAC ambiguities at heterozygous sites, then extracted the focal region from those FASTAs as an alignment using samtools faidx.

## Supplementary information

**Additional file 1: Table S1.** Sample and sequencing information for Brazilian *Heliconius hermathena* and *H. nattereri* generated in this study. **Table S2.***Heliconius hermathena* and *H. nattereri* genome sequencing data. **Table S3.** Samples used for mitochondrial genome assemblies using NOVOplasty (Dierckxens et al., 2017, Nuc Acids Res). **Table S4.** Sample information for other *Heliconius* species used in this study. **Table S5.** Summary statistics of nucleotide diversity per site (pi) and Tajima’s *D* calculated in 100 kb genome-wide. **Table S6.** Numbers of substitutions in *Heliconius nattereri* and relatives. **Table S7.** Numbers of substitutions in *Heliconius hermathena* and relatives. **Table S8.** Whole genome mean *F*_*ST*_ and 95% empirical confidence interval between *H. hermathena* populations. **Table S9.** Whole genome mean *F*_*ST*_ and 95% empirical confidence interval between *H. erato* populations. **Table S10.** Whole genome mean *F*_*ST*_ and 95% empirical confidence interval between *H. melpomene* populations. **Table S11.** Nucleotide diversity, overall and pairwise F_ST_ estimated for *H. hermathena* mitochondrial genomes. **Table S12.** Patterson’s *D* statistics calculated in 10 kb windows across all autosomes. *H. erato* samples are from French Guiana, the geographically closest population to *H. hermathena*.

**Additional file 2: Figure S1.** Guide trees used for progressiveCactus alignments in Edelman et al. (2019) and this study. **Figure S2.** Statistics on unfiltered alignments from the *Heliconius* dataset of 10 kb windows. **Figure S3.** Statistics on unfiltered alignments from the *Heliconius* dataset of coding sequence blocks. Length: length of the raw alignment in base pairs. **Figure S4.** Statistics on unfiltered alignments from the *Heliconius* dataset of non-coding sequence blocks. Length: length of the raw alignment in base pairs. **Figure S5.** Statistics on unfiltered alignments from the *melpomene* clade dataset of 10 kb windows. **Figure S6.** Statistics on unfiltered alignments from the *erato* clade dataset of 10 kb windows. Length: length of the raw alignment in base pairs. **Figure S7**. ASTRAL trees based on filtered a) coding and b) non-coding blocks from *Heliconius* dataset. **Figure S8.** ASTRAL trees based on filtered autosomal 10 kb window alignments from a) the *erato clade* and b) the *melpomene clade*. **Figure S9.** ASTRAL trees based on filtered Z-linked 10 kb window alignments from a) the *erato* clade and b) the *melpomene* clade. **Figure S10.** BUCKy cladograms based on filtered 10 kb window alignments from a) the *erato* clade and b) the *melpomene* clade. **Figure S11.** Predominant topologies for *erato* clade 10 kb autosomal windows. The top 20 topologies accounted for 80% of all windows. **Figure S12.** Historical population size estimates for a) *H. hermathena* and b) *H. nattereri* using PSMC. **Figure S13.** ADMIXTURE analysis of *H. hermathena* polymorphism data for k in 2:10. **Figure S14.** EEMS diversity estimates for *Heliconius hermathena*. **Figure S15.***H. hermathena* Bayesian concordance tree based on 5000 autosomal 10 kb windows, showing all individuals. **Figure S16.** Tests for introgression between *Heliconius hermathena* and *H. charithonia*. **Figure S17.** Divergence (*d*_*xy*_) between *Heliconius sara* and other *erato* clade species in the chromosome 15 inversion and 250 kb flanking regions and ocations of scaffolds that mapped to the inversion breakpoints. **Figure S18.** Convergence of 10 independent EEMS runs.

## Data Availability

Genome assemblies and sequencing data generated in this study are available at the NCBI associated with BioProjects PRJNA596795 [103] and PRJNA596801 [104]. Genome assemblies are available at the NCBI under BioProjects PRJNA596796 [105] and PRJNA596794 [106].
