# Supplementary material for: The roles of hybridization and habitat fragmentation in the evolution of Brazil’s enigmatic longwing butterflies, Heliconius nattereri and H. hermathena
Source: BMC Biol. 2020 Jul 3;18:84. doi: 10.1186/s12915-020-00797-1 (PMC7334841; doi:10.1186/s12915-020-00797-1)
Supplement: Supplementary file 2 — Additional file 2: Figure S1. Guide trees used for progressiveCactus alignments in Edelman et al. (2019) and this study. Figure S2. Statistics on unfiltered alignments from the Heliconius dataset of 10 kb windows. Figure S3. Statistics on unfiltered alignments from the Heliconius dataset of coding sequence blocks. Length: length of the raw alignment in base pairs. Figure S4. Statistics on unfiltered alignments from the Heliconius dataset of non-coding sequence blocks. Length: length of the raw alignment in base pairs. Figure S5. Statistics on unfiltered alignments from the melpomene clade dataset of 10 kb windows. Figure S6. Statistics on unfiltered alignments from the erato clade dataset of 10 kb windows. Length: length of the raw alignment in base pairs. Figure S7. ASTRAL trees based on filtered a) coding and b) non-coding blocks from Heliconius dataset. Figure S8. ASTRAL trees based on filtered autosomal 10 kb window alignments from a) the erato clade and b) the melpomene clade. Figure S9. ASTRAL trees based on filtered Z-linked 10 kb window alignments from a) the erato clade and b) the melpomene clade. Figure S10. BUCKy cladograms based on filtered 10 kb window alignments from a) the erato clade and b) the melpomene clade. Figure S11. Predominant topologies for erato clade 10 kb autosomal windows. The top 20 topologies accounted for 80% of all windows. Figure S12. Historical population size estimates for a) H. hermathena and b) H. nattereri using PSMC. Figure S13. ADMIXTURE analysis of H. hermathena polymorphism data for k in 2:10. Figure S14. EEMS diversity estimates for Heliconius hermathena. Figure S15.H. hermathena Bayesian concordance tree based on 5000 autosomal 10 kb windows, showing all individuals. Figure S16. Tests for introgression between Heliconius hermathena and H. charithonia. Figure S17. Divergence (dxy) between Heliconius sara and other erato clade species in the chromosome 15 inversion and 250 kb flanking regions and ocations of scaffolds that [file 12915_2020_797_MOESM2_ESM.pdf]

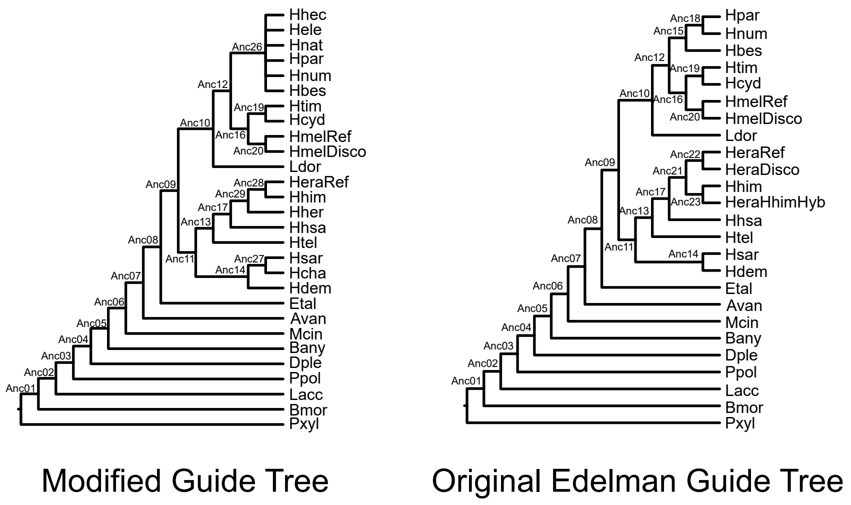

Figure S1. Guide trees used for progressiveCactus alignments in Edelman et al. (2019) and this study.

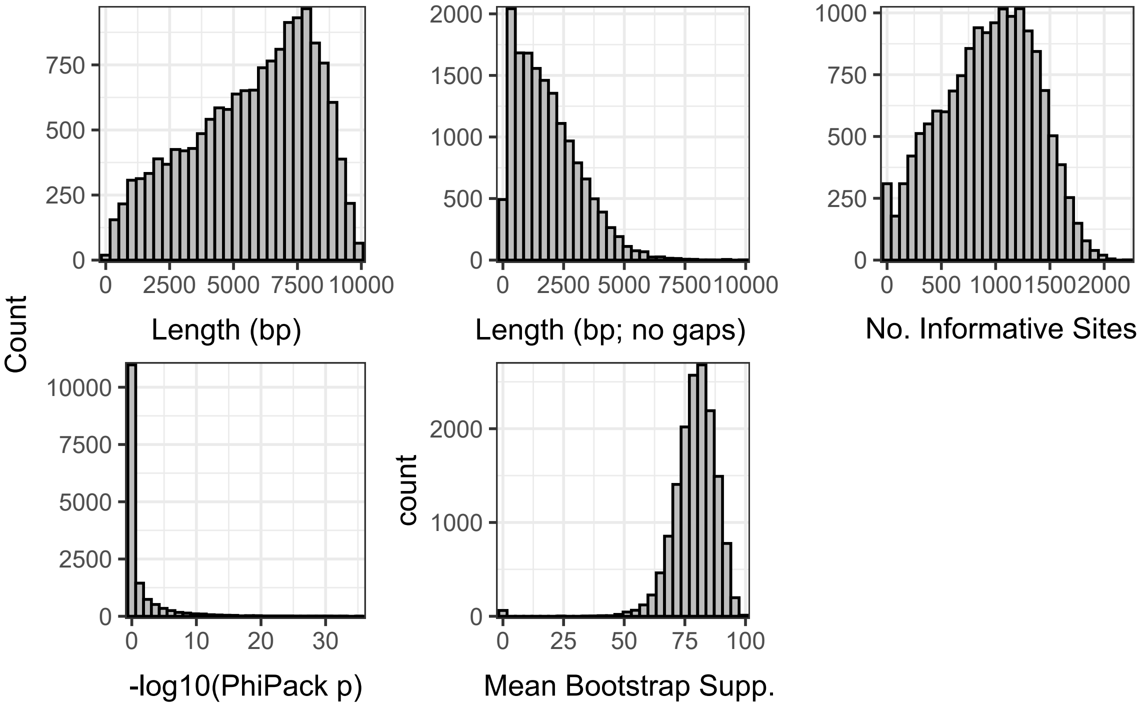

Figure S2. Statistics on unfiltered alignments from the *Heliconius* dataset of 10 kb windows. Length: length of the raw alignment in base pairs. Length (no gaps): length of the alignment after stripping all columns with a gap in any species. No. Informative Sites: number of informative sites in the ungapped alignment. -log(PhiPack *p*): statistical test *p*-value for assessing recombination in the ungapped alignment. Mean Bootstrap Supp.: Average bootstrap support over all nodes in the maximum likelihood tree.

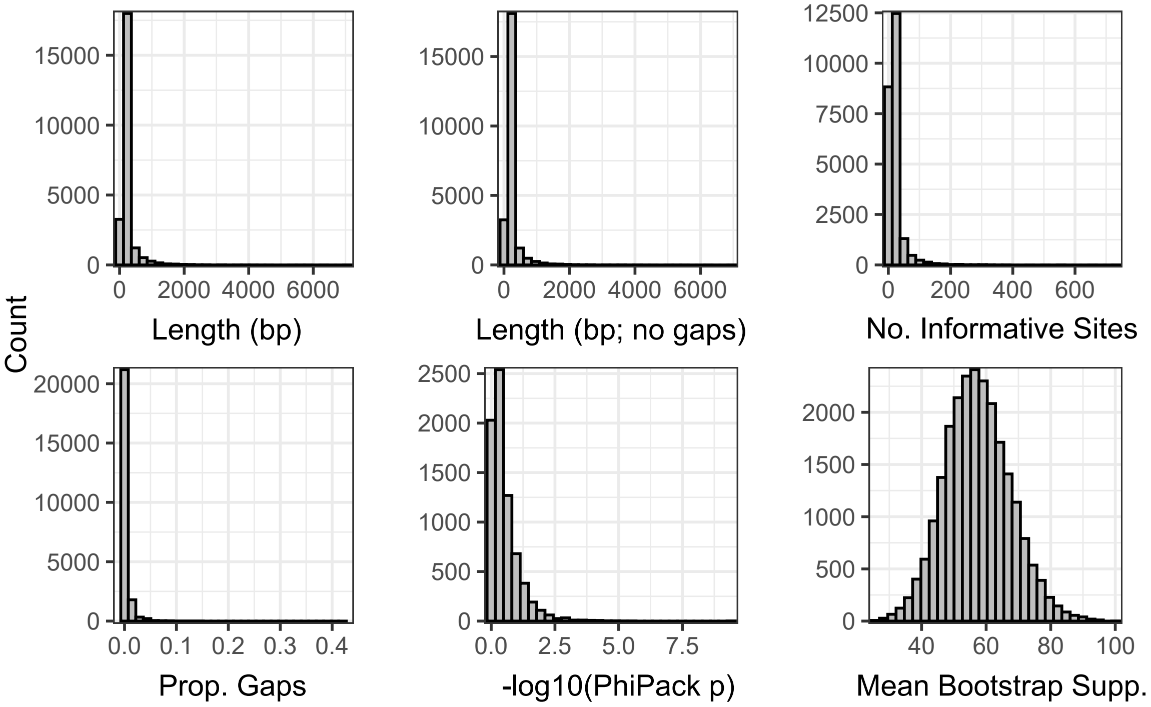

Figure S3. Statistics on unfiltered alignments from the *Heliconius* dataset of coding sequence blocks. Length: length of the raw alignment in base pairs. Length (no gaps): length of the alignment after stripping all columns with a gap in any species. No. Informative Sites: number of informative sites in the ungapped alignment. -log(PhiPack *p*): statistical test *p*-value for assessing recombination in the ungapped alignment. Mean Bootstrap Supp.: Average bootstrap support over all nodes in the maximum likelihood tree.

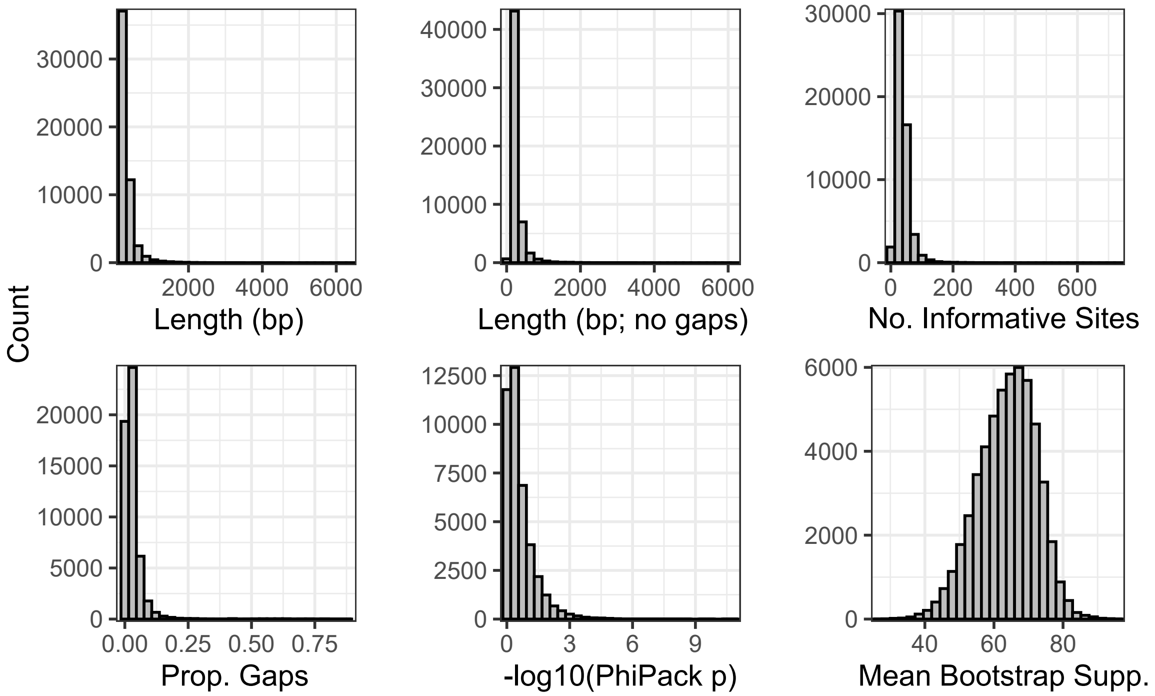

Figure S4. Statistics on unfiltered alignments from the *Heliconius* dataset of non-coding sequence blocks. Length: length of the raw alignment in base pairs. Length (no gaps): length of the alignment after stripping all columns with a gap in any species. No. Informative Sites: number of informative sites in the ungapped alignment. -log(PhiPack *p*): statistical test *p*-value for assessing recombination in the ungapped alignment. Mean Bootstrap Supp.: Average bootstrap support over all nodes in the maximum likelihood tree.

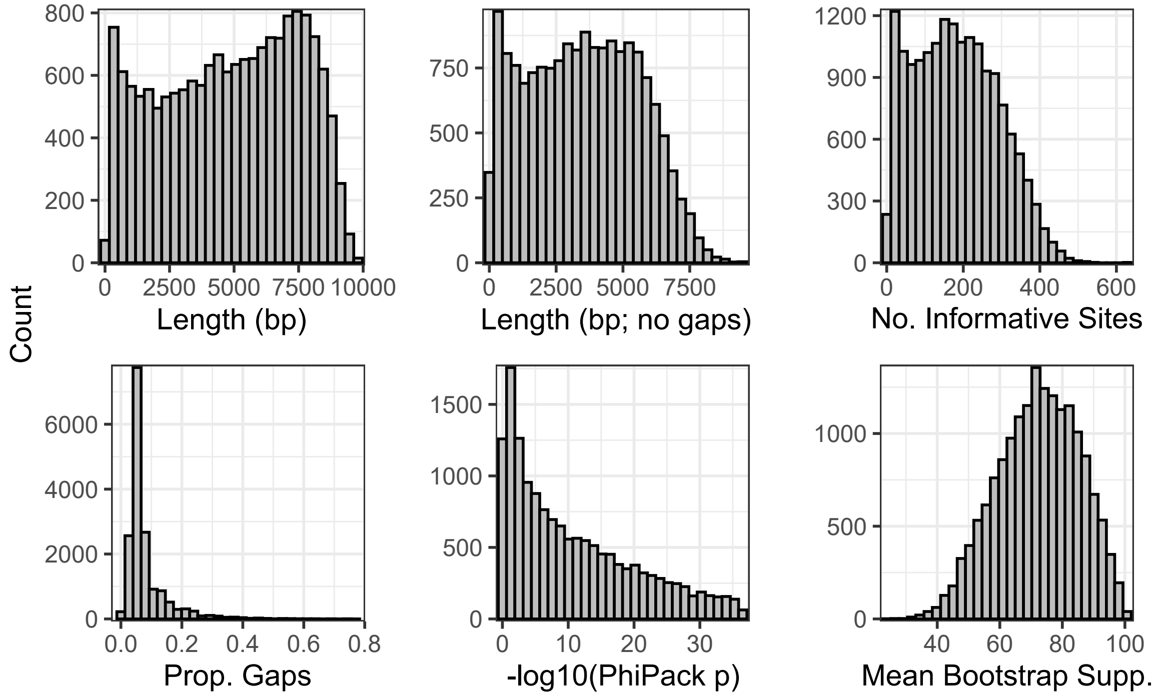

Figure S5. Statistics on unfiltered alignments from the *melpomene* clade dataset of 10 kb windows. Length: length of the raw alignment in base pairs. Length (no gaps): length of the alignment after stripping all columns with a gap in any species. No. Informative Sites: number of informative sites in the ungapped alignment. -log(PhiPack *p*): statistical test *p*-value for assessing recombination in the ungapped alignment. Mean Bootstrap Supp.: Average boots trap support over all nodes in the maximum likelihood tree.

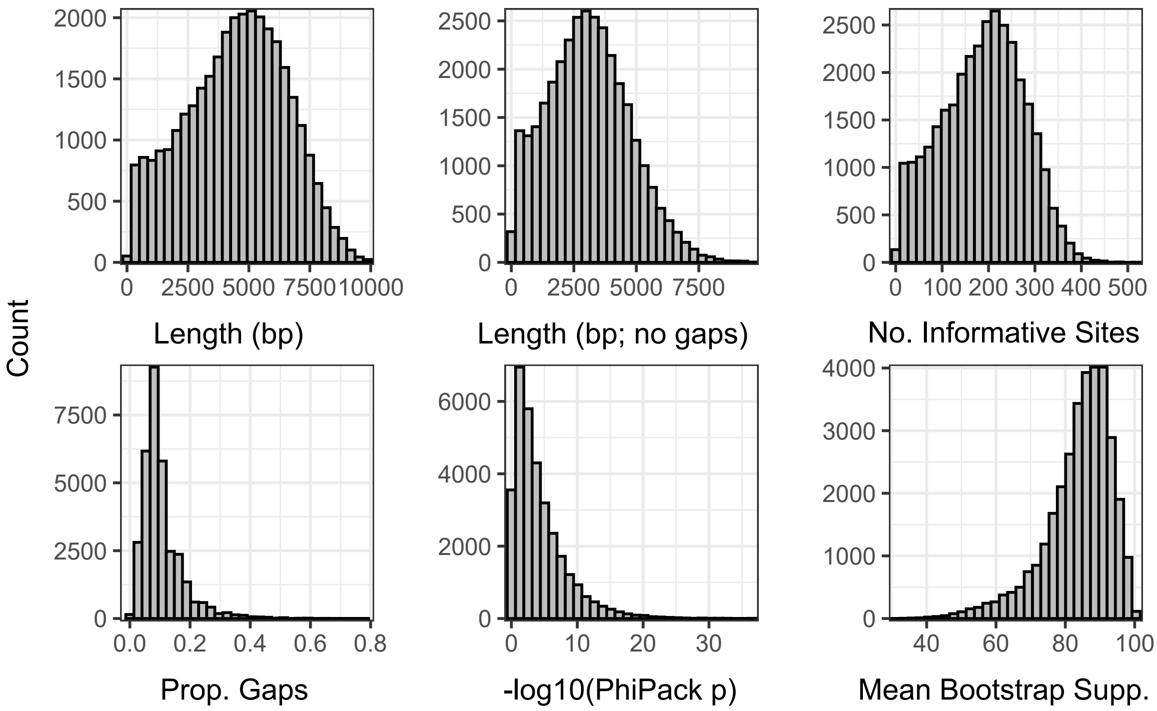

Figure S6. Statistics on unfiltered alignments from the *erato* clade dataset of 10 kb windows. Length: length of the raw alignment in base pairs. Length (no gaps): length of the alignment after stripping all columns with a gap in any species. No. Informative Sites: number of informative sites in the ungapped alignment. -log(PhiPack *p*): statistical test *p*-value for assessing recombination in the ungapped alignment. Mean Bootstrap Supp.: Average boot strap support over all nodes in the maximum likelihood tree.

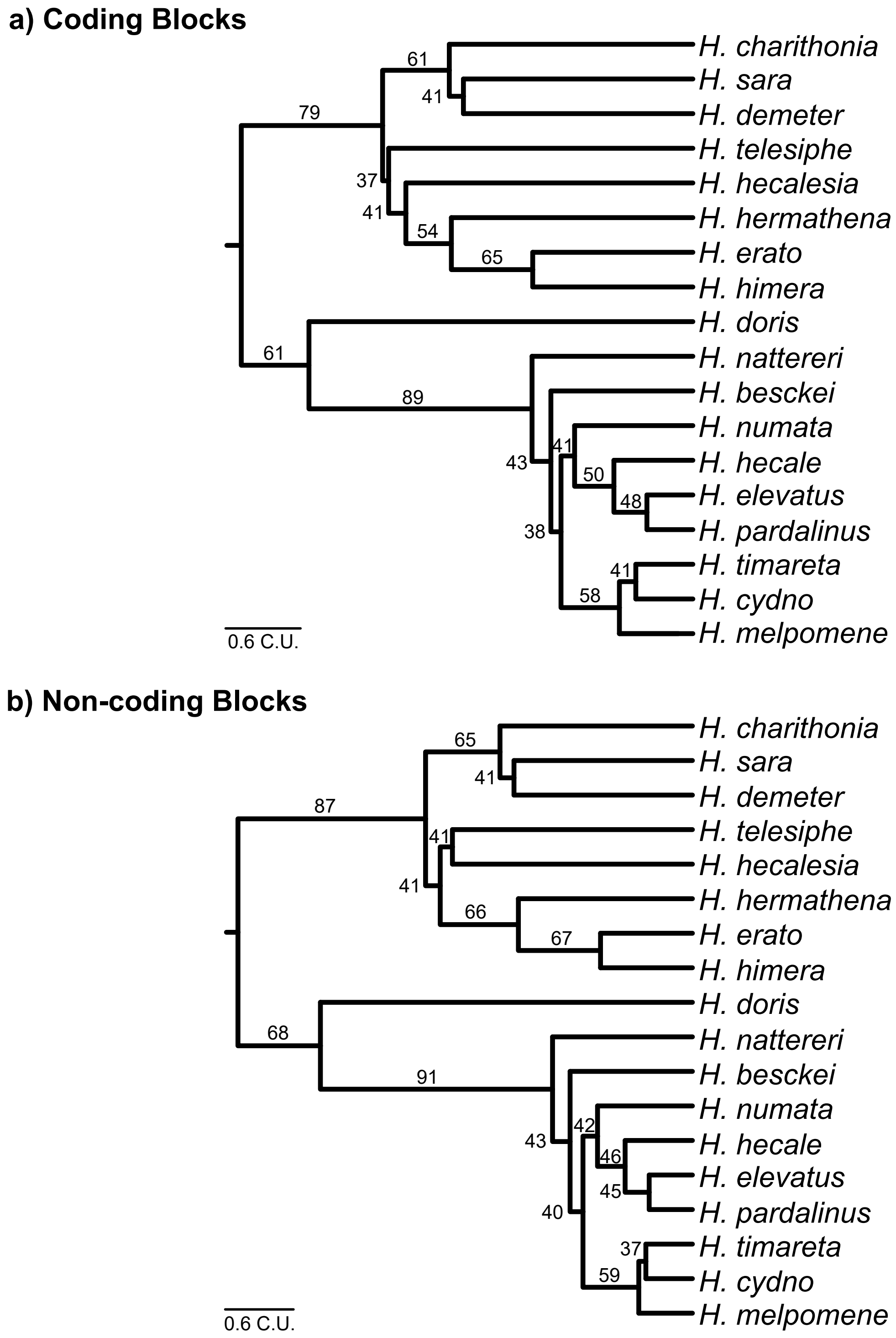

Figure S7. ASTRAL trees based on filtered **a)** coding and **b)** non-coding blocks from *Heliconius* dataset. Trees were rooted using *Eueides tales* (not shown). The values above branches are ASTRAL quartet scores. Terminal branch lengths are meaningless, but internal branch lengths are scaled to coalescent units (C.U.).

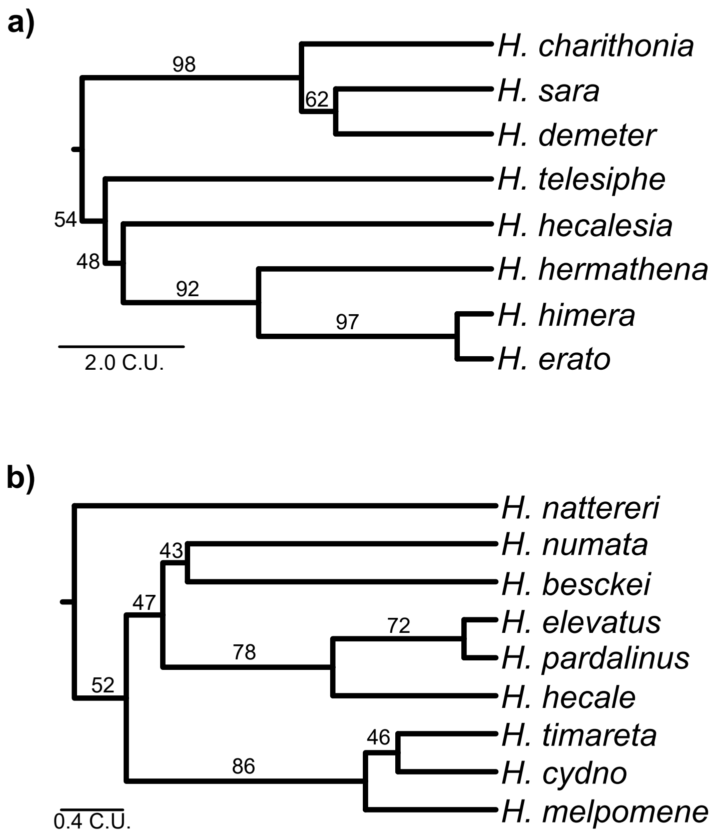

Figure S8. ASTRAL trees based on filtered autosomal 10 kb window alignments from **a)** the *erato* clade and **b)** the *melpomene* clade. Trees were rooted using *H. melpomene* or *H. erato,* respectively (not shown). The values above branches are ASTRAL quartet scores. Terminal branch lengths are meaningless, but internal branch lengths are scaled to coalescent units (C.U.).

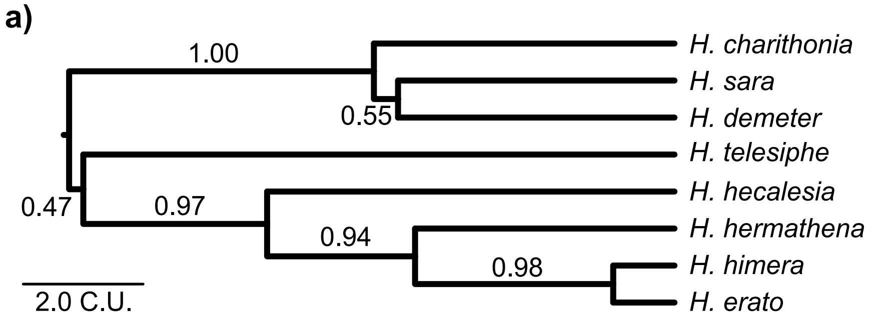

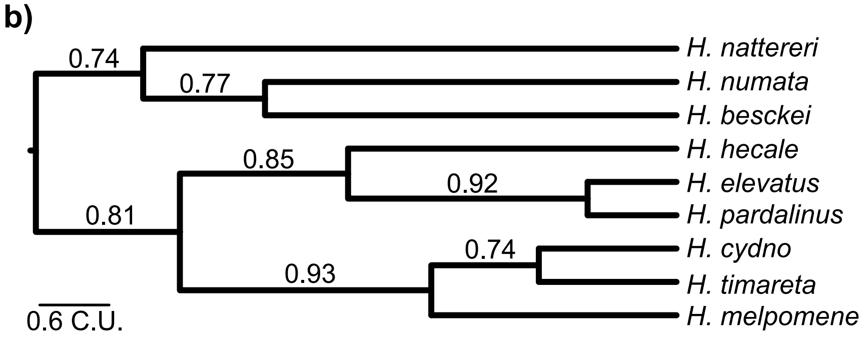

Figure S9. ASTRAL trees based on filtered Z-linked 10 kb window alignments from **a)** the *erato* clade and **b)** the *melpomene* clade. Trees were rooted using *H. melpomene* or *H. erato,* respectively (not shown). The values above branches are ASTRAL quartet scores. Terminal branch lengths are meaningless, but internal branch lengths are scaled to coalescent units (C.U.).

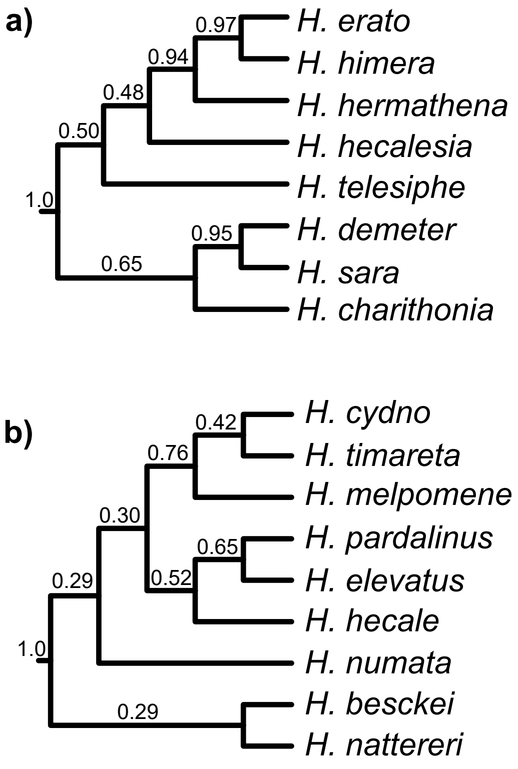

Figure S10. BUCKy cladograms based on filtered 10 kb window alignments from **a)** the *erato* clade and **b)** the *melpomene* clade. Trees were rooted using *H. melpomene* or *H. erato,* respectively (not shown). The values above branches are Bayesian concordance factors.

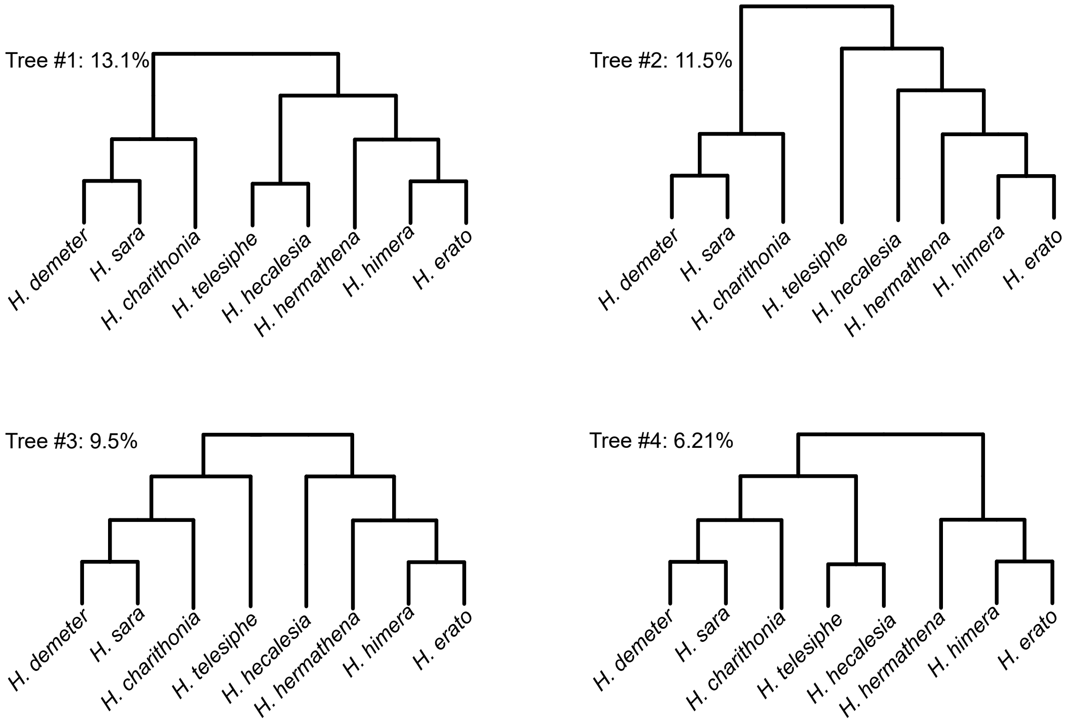

Figure S11. Predominant topologies for *erato* clade 10 kb autosomal windows. The top 20 topologies accounted for 80% of all windows.

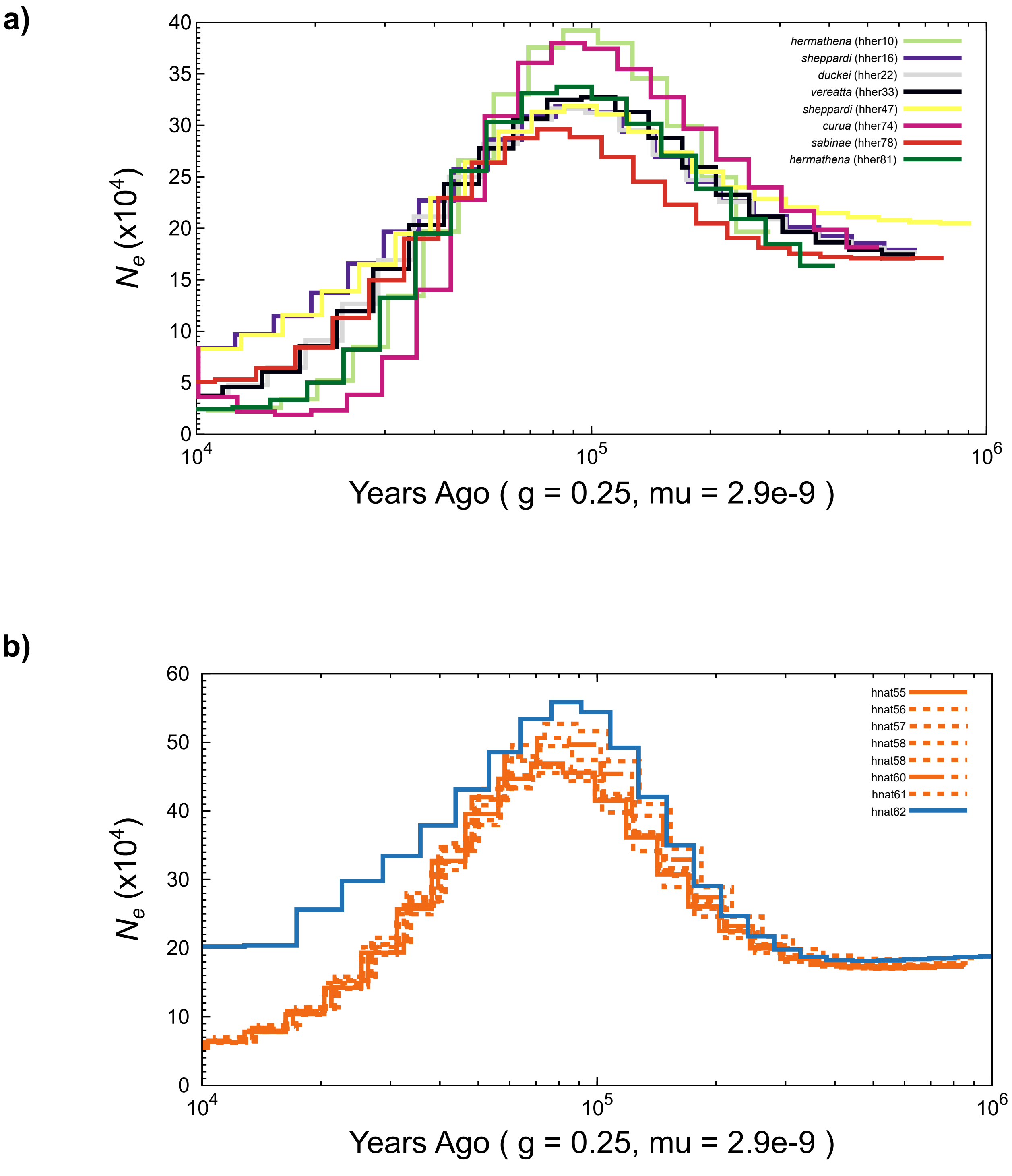

Figure S12. Historical population size estimates for **a)** *H. hermathena* and **b)** *H. nattereri* using PSMC. Colors follow Fig. 1a. g: generations per year, mu: mutation rate per bp per year.

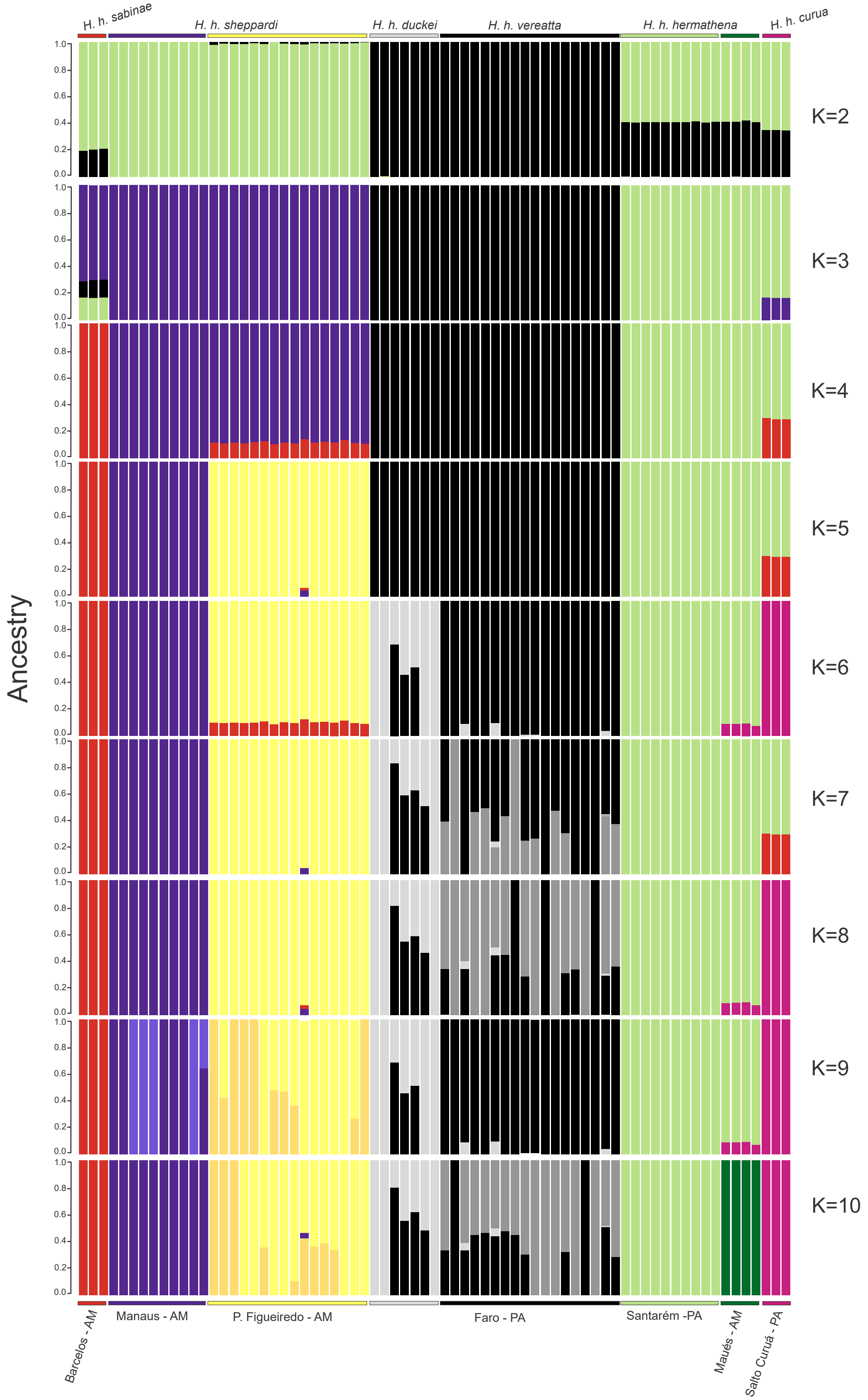

Figure S13. ADMIXTURE analysis of *H. hermathena* polymorphism data for *k* in 2:10.

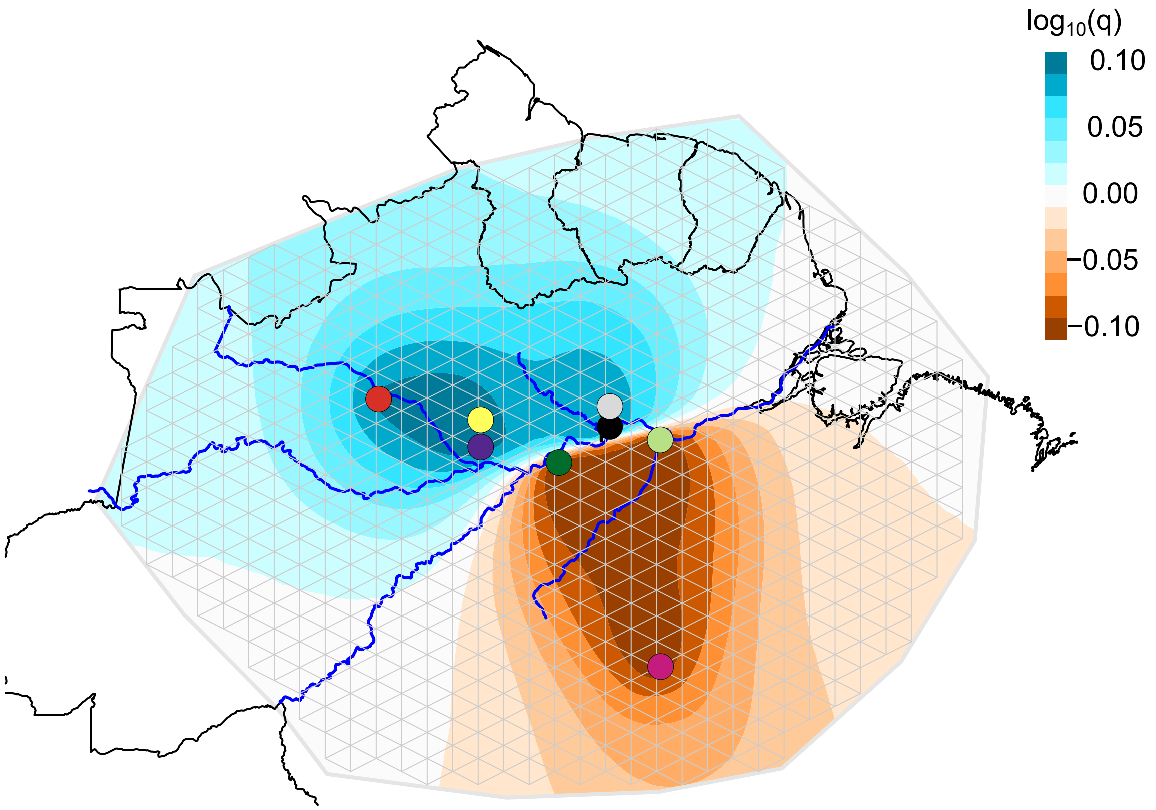

Figure S14. EEMS diversity estimates for *Heliconius hermathena.* q: relative genetic diversity.

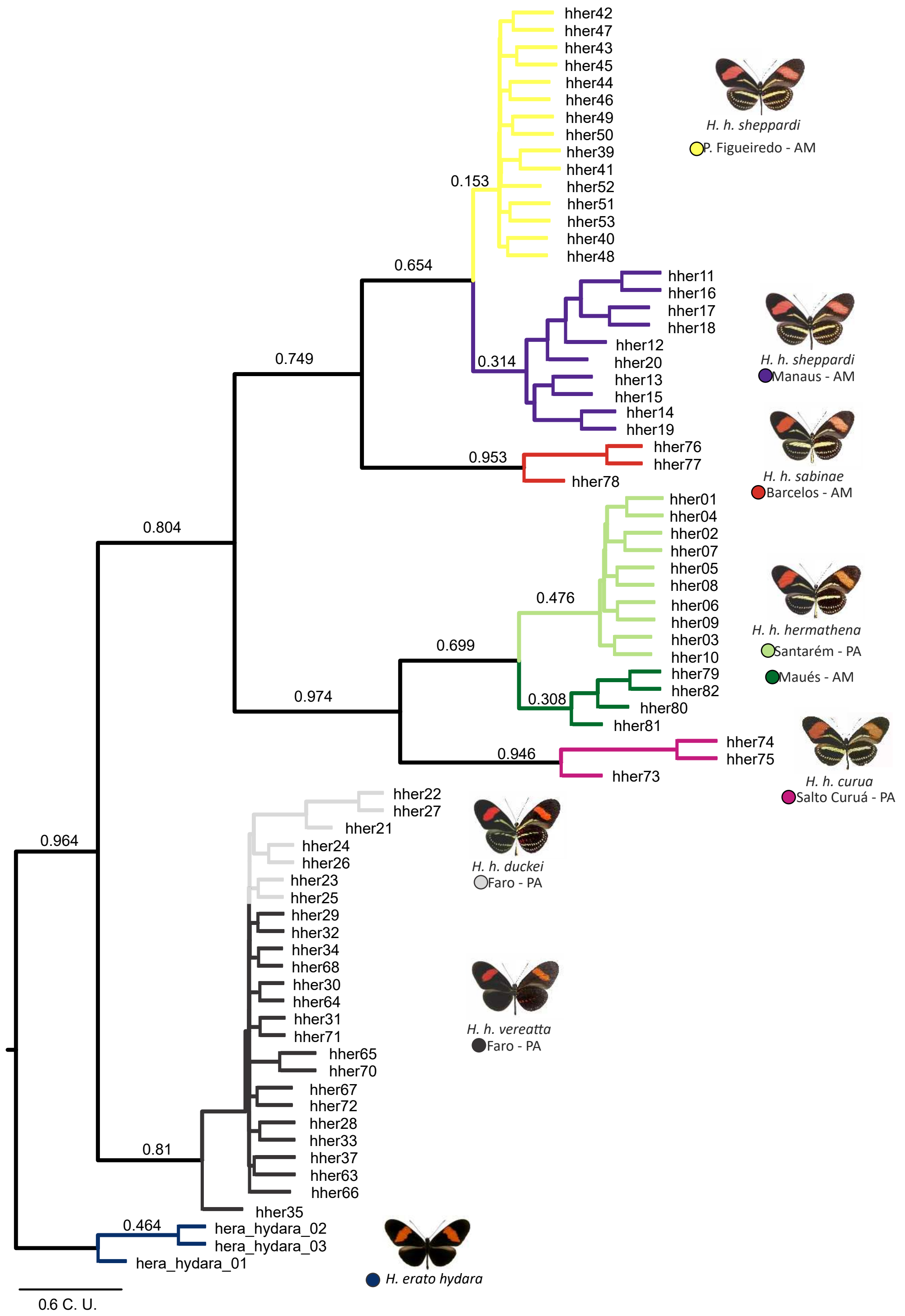

Figure S15. *H. hermathena* Bayesian concordance tree based on 5,000 autosomal 10 kb windows, showing all individuals. Concordance factors are shown only for the major clades. PA: Para, AM: Amazonas.

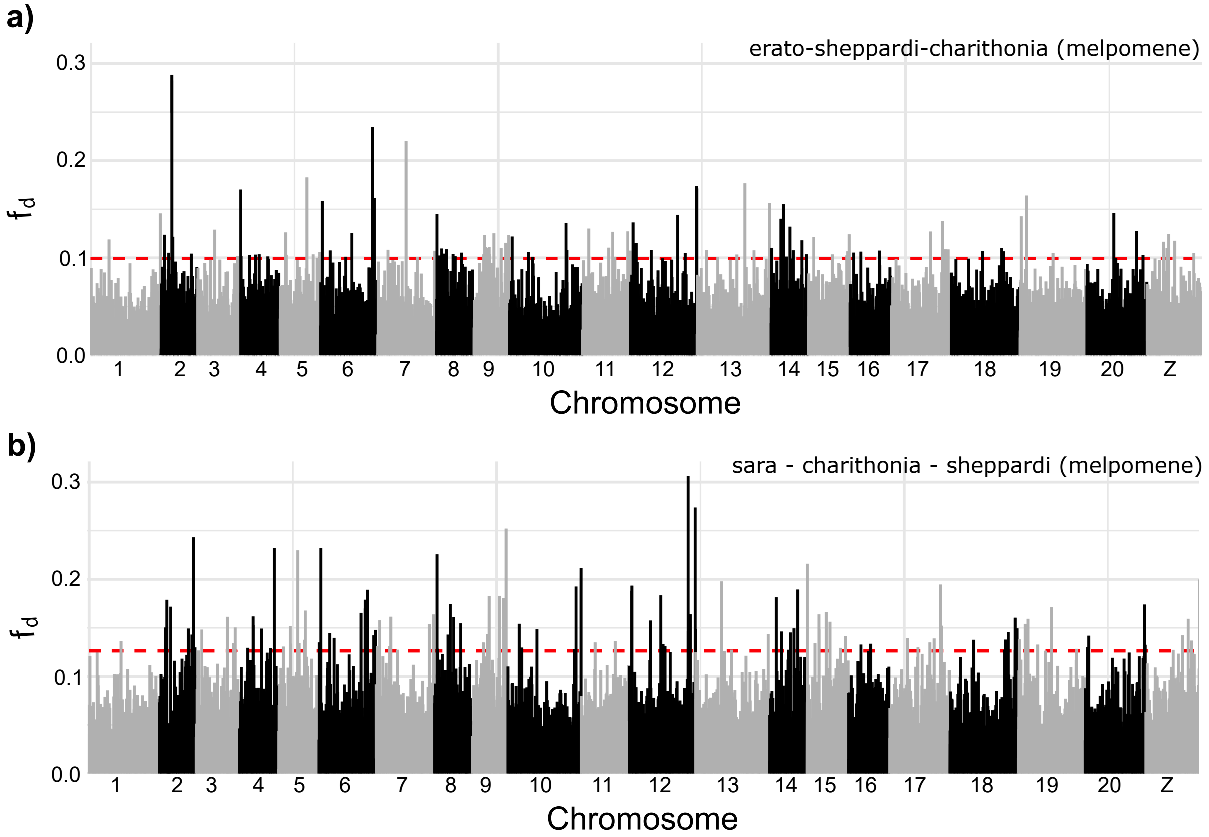

Figure S16. Tests for introgression between *Heliconius hermathena* and *H. charithonia*. f_d was calculated in non-overlapping 10 kb windows. Comparisons are shown as P1-P2-P3-(outgroup).

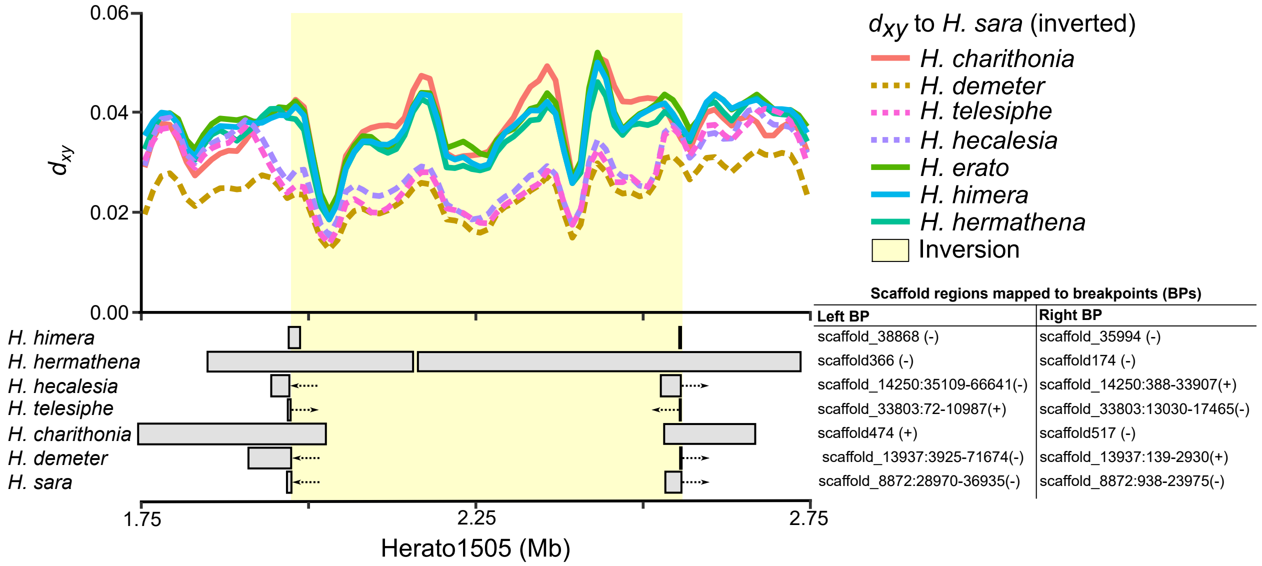

Scaffold regions mapped to breakpoints (BPs)

	Left BP	Right BP
H. himera	scaffold_38868 (-)	scaffold_35994 (-)
H. hermathena	scaffold366 (-)	scaffold174 (-)
H. hecalesia	scaffold_14250:35109-66641(-)	scaffold_14250:388-33907(+)
H. telesiphe	scaffold_33803:72-10987(+)	scaffold_33803:13030-17465(-)
H. charithonia	scaffold474 (+)	scaffold517 (-)
H. demeter	scaffold_13937:3925-71674(-)	scaffold_13937:139-2930(+)
H. sara	scaffold_8872:28970-36935(-)	scaffold_8872:938-23975(-)

Figure S17. Top: Divergence (d_{xy}) between *Heliconius sara* and other *erato* clade species in the chromosome 15 inversion and 250 kb flanking regions. Lines are loess best fits based on d_{xy} calculated non-overlapping 10 kb windows from the whole genome alignment. Bottom: Locations of scaffolds that mapped to the inversion breakpoints. For each species, we pulled all scaffolds that mapped to within 500 kb of the putative inversion breakpoints, then mapped them to the *H. erato* reference genome using BLAST. For each species, we represent the scaffold or scaffolds that span the breakpoints as gray bars under the locations they map to in the *H. erato* genome. The mapped regions of the scaffolds are shown in the table to the right (scaffold:start-end(orientation)). Dashed arrows indicate the direction of mapping.

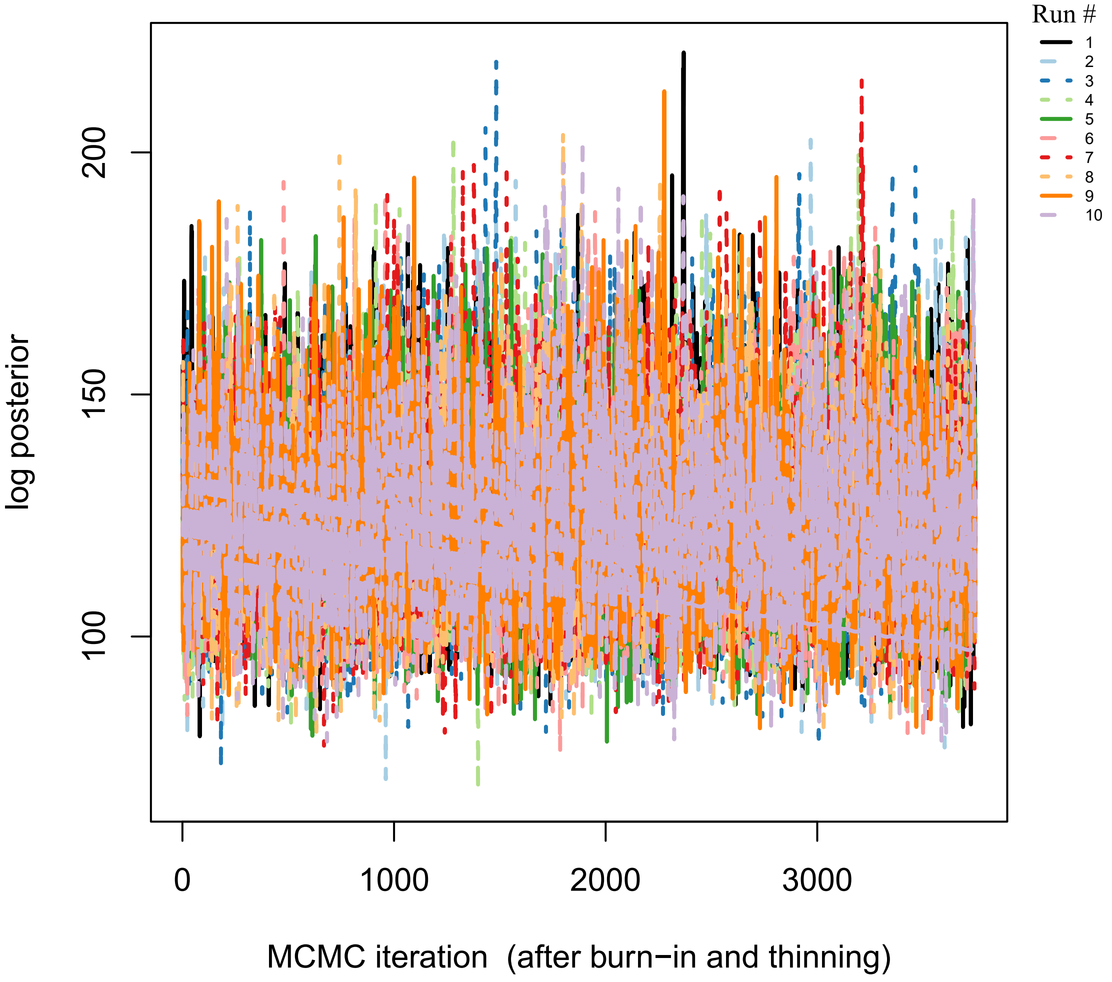

Figure S18. Convergence of 10 independent EEMS runs.
